# Continuing evolution of H6N2 influenza a virus in South African chickens and the implications for diagnosis and control

**DOI:** 10.1186/s12917-019-2210-4

**Published:** 2019-12-18

**Authors:** Celia Abolnik, Christine Strydom, Dionne Linda Rauff, Daniel Barend Rudolph Wandrag, Deryn Petty

**Affiliations:** 1 Department of Production Animal Studies, Faculty of Veterinary Science, University of Pretoria, Old Soutpan Road, Onderstepoort, 0110 South Africa; 2 Deltamune (Pty) Ltd, 248 Jean Avenue, Lyttleton, Centurion, 0140 South Africa; 3 The Poultry Practice, PO Box 5615, Walmer, Port Elizabeth, 6065 South Africa

**Keywords:** H6N2 avian influenza, Chickens, Evolution, Human markers, Serological diagnosis

## Abstract

**Background:**

The threat of poultry-origin H6 avian influenza viruses to human health emphasizes the importance of monitoring their evolution. South Africa’s H6N2 epidemic in chickens began in 2001 and two co-circulating antigenic sub-lineages of H6N2 could be distinguished from the outset. The true incidence and prevalence of H6N2 in the country has been difficult to determine, partly due to the continued use of an inactivated whole virus H6N2 vaccine and the inability to distinguish vaccinated from non-vaccinated birds on serology tests. In the present study, the complete genomes of 12 H6N2 viruses isolated from various farming systems between September 2015 and February 2019 in three major chicken-producing regions were analysed and a serological experiment was used to demonstrate the effects of antigenic mismatch in diagnostic tests.

**Results:**

Genetic drift in H6N2 continued and antigenic diversity in sub-lineage I is increasing; no sub-lineage II viruses were detected. Reassortment patterns indicated epidemiological connections between provinces as well as different farming systems, but there was no reassortment with wild bird or ostrich influenza viruses. The sequence mismatch between the official antigens used for routine hemagglutination inhibition (HI) testing and circulating field strains has increased steadily, and we demonstrated that H6N2 field infections are likely to be missed. More concerning, sub-lineage I H6N2 viruses acquired three of the nine HA mutations associated with human receptor-binding preference (A13S, V187D and A193N) since 2002. Most sub-lineage I viruses isolated since 2015 acquired the K702R mutation in PB2 associated with the ability to infect humans, whereas prior to 2015 most viruses in sub-lineages I and II contained the avian lysine marker. All strains had an unusual HA_0_ motif of PQVETRGIF or PQVGTRGIF.

**Conclusions:**

The H6N2 viruses in South African chickens are mutating and reassorting amongst themselves but have remained a genetically pure lineage since they emerged more than 18 years ago. Greater efforts must be made by government and industry in the continuous isolation and characterization of field strains for use as HI antigens, new vaccine seed strains and to monitor the zoonotic threat of H6N2 viruses.

## Background

Avian influenza A virus (IAV) serotypes are formed by the combination of two major antigens on the viral surface, namely hemagglutinin protein (H) of which 16 subtypes have been identified, and neuraminidase (N) protein of which nine subtypes are known. The natural propensity of IAV to reassort its eight genomic RNA segments during co-infection can lead to H1 to H16 and N1 to N9 occurring in virtually any combination, but serotypes have a great range in host specificity and pathogenic potential [1]. The disease “avian influenza” is defined in the World Organization for Animal Health (OIE) Terrestrial Animal Health Code as an infection of poultry caused by any influenza A virus with high pathogenicity (HPAI) (i.e. an intra-venous pathogenicity index of > 1.2 in chickens), and by H5 and H7 subtypes with low pathogenicity (LPAI) [2]. Only viruses in the aforementioned categories are reportable to the OIE by member states and the control of other subtypes in poultry is left to the discretion of the country. Should a country opt to use vaccination as a means of control or reducing infections, the OIE recommends that vaccine strains be re-evaluated at least every 2 to 3 years for efficacy against circulating field viruses and updated as required. Periodically evaluating and updating diagnostic tests to keep abreast of genetic and antigenic drift in IAV is equally important.

H6 is one of the most common subtypes detected in the migratory waterfowl reservoir, but most H6 viruses introduced from waterfowl into domestic poultry have been restricted in their ability to spread. However, since 2001 H6N2 circulated in meat turkey flocks in Germany [3], H6N1 infected meat turkeys in France [4] and H6N2 caused sustained outbreaks among domestic poultry in the USA from 2000 to 2005 [5, 6]. H6 IAVs have been one of the predominant subtypes circulating since the 1970’s in live bird markets in southern China and later South Korea, Vietnam, Cambodia, Thailand and Laos. In the East Asian sub-region, H6N1, H6N2, H6N5, H6N6, H6N8 and H6N9 co-circulate in chicken and duck populations, undergoing frequent reassortment with other subtypes [7–12].

H6 has a broader host range than any other IAV subtype. Its ability to replicate well in mice without pre-adaptation was an early indication that these viruses could cause infections in mammals [13, 14]. H6 subtypes have since been isolated from cases of respiratory illness in pigs [15, 16] and dogs [17]. Serologic surveillance of humans revealed that veterinarians and agricultural workers exposed to H6 IAV-infected poultry could also become infected [18, 19] and in May 2013 the first human infection with an avian influenza H6 subtype linked to respiratory illness was reported in Taiwan. In that case a poultry-origin H6N1 virus was isolated from a 20-year-old woman with influenza-like symptoms who recovered, but there was no evidence of human-to-human transmission [20]. The threat of H6 AIVs to human health emphasizes the importance of continuous surveillance for H6 IAVs in poultry, and alarmingly up to 34% of H6 IAVs identified in Chinese poultry contained molecular markers that were demonstrated to enable binding to human type receptors [21].

**Fig. 1:**
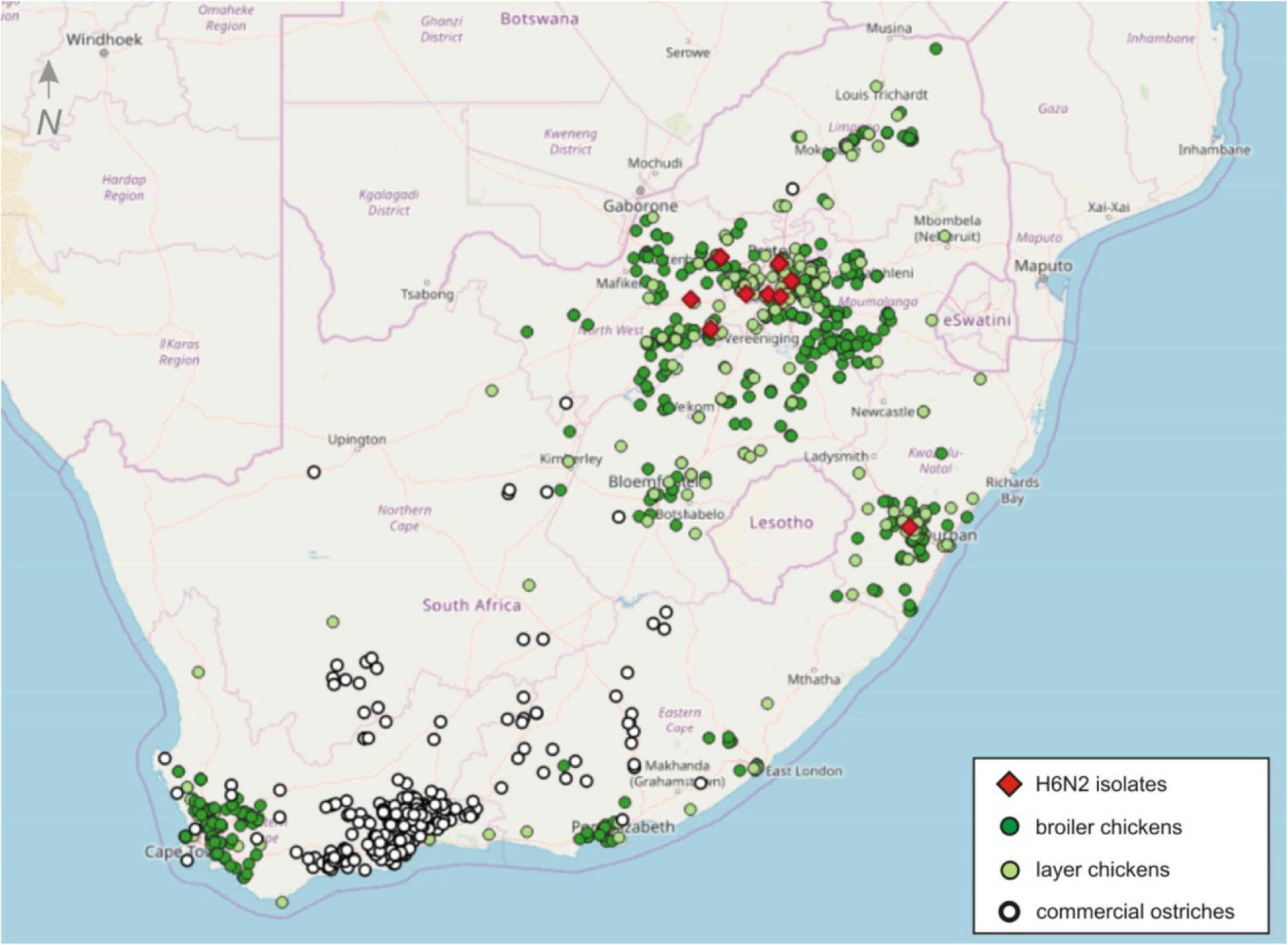
Registered poultry farming operations in South Africa and locations from which the H6N2 isolates in this study were obtained. The map was produced in QGIS v3.8 (https://qgis.org/en/site/)

South Africa’s H6N2 epidemic in chickens began in 2001. The progenitor was traced to a reassortment between viruses that infected commercial ostriches in their major production area of the Western Cape Province in the mid to late 1990’s (Fig. 1) notably an H6N8 virus and an H9N2 virus [22]. Personal communications from the veterinarians involved cited an outbreak in a commercial layer farm in the Western Cape Province with the spent hens subsequently transported by a cull buyer to the KwaZulu Natal (KZN) province. Once the chickens in the villages of poultry workers in Camperdown area of KZN became infected, poor biosecurity on the nearby commercial farms soon led to the first outbreaks in the intensive operations. After that the H6N2 infection spread rapidly along transportation networks to other provinces [22]. Two antigenic sub-lineages of H6N2 could be distinguished from the outset where sub-lineage I appeared to be derived from sub-lineage II and is characterized by additional chicken-specific adaptations such as an NA-stalk deletion and hyper N-glycosylated HA head [22]. Both sub-lineages co-circulated up until at least 2013 [23].

Locally, clinical signs of H6N2 in layers range from nearly inapparent to quite severe respiratory signs with egg production drops of up to 30%. Weekly mortality increases between 1.6 and 5.1-fold and the hens take between 4 and 6 weeks to recover production. Severe losses in broiler breeders are also reported, with production drops of between 5 and 60% and with increased mortalities of up to 37% but concomitant infections with other respiratory pathogens such as *Mycoplasma gallisepticum* often influence the disease severity. An inactivated whole virus vaccine prepared from a 2002 sub-lineage I seed strain has been applied, under permission of the state and with strict conditions, to protect birds against clinical disease, but the continued use of this vaccine was previously shown to accelerate the antigenic drift within sub-lineage I isolates [23].

In South Africa H6N2 is a controlled disease and is thus included in the compulsory monthly serological testing of registered compartmentalised commercial poultry operations and compulsory six-monthly serological testing of all other commercial poultry farms plus a statistically significant sample of non-commercial poultry flocks (e.g. backyard chickens). Each epidemiological unit is tested to detect infection at > 10% prevalence with 95% confidence [24]. Sera are screened using validated commercial influenza A antibody ELISA tests, and positive samples are subsequently tested using hemagglutination inhibition (HI) assays against the H5, H6 and H7 subtypes. Positive results are followed up with additional sampling, including the collection of swabs for viral detection. The true incidence and prevalence of H6N2 has been difficult to determine, partly due to the continued use of an inactivated whole virus H6N2 vaccine and the inability to distinguish vaccinated from non-vaccinated birds on serology. The placement of marked unvaccinated sentinels is prescribed to enable the diagnosis of field challenge where the vaccine is applied. Occasionally, veterinarians will submit swabs or tissues from mortalities where H6N2 infection is suspected for isolation, but this is not done routinely. In the present study, 12 H6N2 viruses isolated from commercial chickens in South Africa from 2015 to 2019 were analysed for genetic and antigenic drift, and for markers associated with the ability to infect humans. Furthermore, a serological experiment was used to demonstrate the effects of antigenic mismatch in diagnostic tests.

## Results

### Virus isolation

Twelve H6N2 viruses were isolated from various farming systems between September 2015 and February 2019, in three major chicken-producing regions in South Africa (Fig. 1), namely Gauteng (*n* = 7), North West (*n* = 4) and Kwa-Zulu Natal (*n* = 1) Provinces (Table 1). No viruses were isolated from the Western Cape or other provinces during this period, although serological test results suggest that infection is widespread (unpublished national veterinary laboratory records). Viruses isolated in 2015 showed normal patterns of agglutination of chicken red blood cells (RBCs) on the HI tests. Viruses isolated in 2016 showed either weak agglutination or no agglutination on the first passage but dwarfing of the embryos and deaths was observed. Similarly, isolate 432/2019 did not agglutinate RBCs on the first passage, but the allantoic fluid tested positive for IAV on rRT-PCR and H6 conventional RT-PCR (results not shown).

**Table 1:** H6N2 viruses isolated during the study

Strain	Collection date	Location	Host	GenBank accession numbers
338087^a^	17 Sep 2015	Johannesburg, GAU	Broilers, 27 days	MK996153-MK996160
341797^a^	9 Oct 2015	Rustenburg, NW	Broiler breeders, age unknown	MK996169-MK996176
339678^a^	13 Oct 2015	Rustenburg, NW	Broiler breeders, 43 weeks	MK996161-MK996168
344378^a^	13 Oct 2015	Bapsfontein, GAU	Layers, 47 weeks	MK996177-MK996184
344579^a^	12 Oct 2015	Bapsfontein, GAU	Layers, 37 weeks	MK996185-MK996192
398997^a^	11 Oct 2016	Boksburg, GAU	Layers, 7 weeks	MK996193-MK996200
N2826^b^	8 Jul 2016	Pretoria, GAU	Broilers, 21 days	MH170286-MH170293
401156^a^	9 Oct 2016	Ventersdorp, NW	Layers, age unknown	MK996201-MK996208
402385^a^	24 Oct 2016	Potchefstroom, NW	Broiler breeders, 63 weeks	MK996209-MK996216
404573^a^	9 Nov 2016	Randfontein, GAU	Layers, age unknown	MK996217-MK996224
H44954^c^	1 Nov 2016	Pietermaritzburg, KZN	Layers, 56 weeks	MK996225-MK996232
432^a,b^	13 Feb 2019	Pretoria, GAU	Layers, 20 weeks	MK996233-MK996240

*GAU* Gauteng Province, *NW* North West Province, *KZN* KwaZulu-Natal Province

^a^Isolated at Deltamune laboratories (Pty) Ltd

^b^Isolated at the University of Pretoria

^c^Isolated at RCL Foods

### Genome assembly, phylogenetic and reassortment analysis

**Fig. 2:**
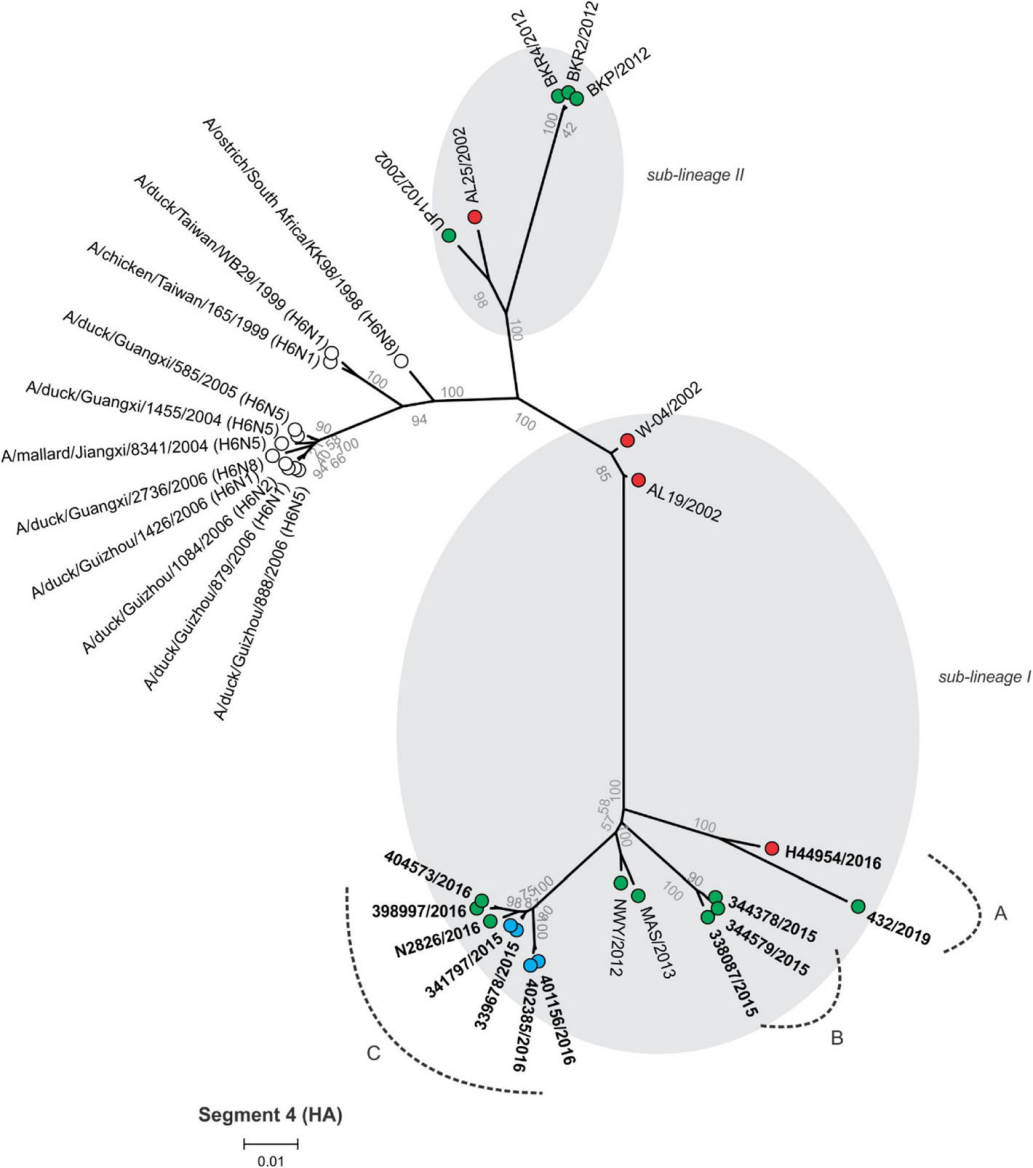
Maximum likelihood phylogenetic tree of segment 4 encoding the hemagglutinin (HA) protein gene of South African H6N2 chicken and reference viruses. Green = Gauteng Province; Blue = North West Province; Red = KwaZulu-Natal Province; clear = reference strains. Viruses sequenced in the present study are in boldface. Genetic clusters are labelled A, B and C

**Fig. 3:**
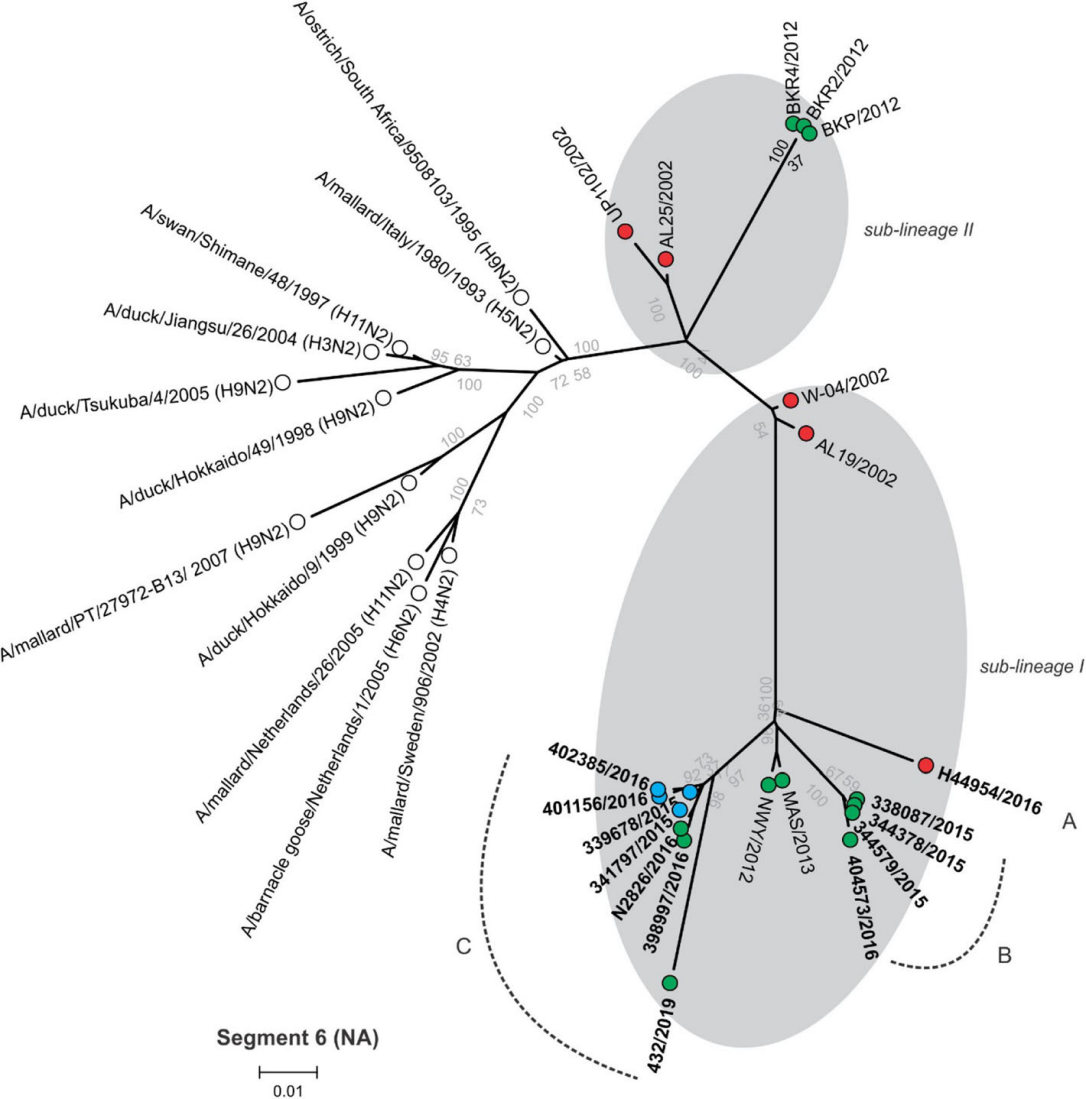
Maximum likelihood phylogenetic tree of segment 6 encoding the neuraminidase (NA) protein of South African H6N2 chicken and reference viruses. Green = Gauteng Province; Blue = North West Province; Red = KwaZulu-Natal Province; clear = reference strains. Viruses sequenced in the present study are in boldface. Genetic clusters are labelled A, B and C

**Fig. 4:**
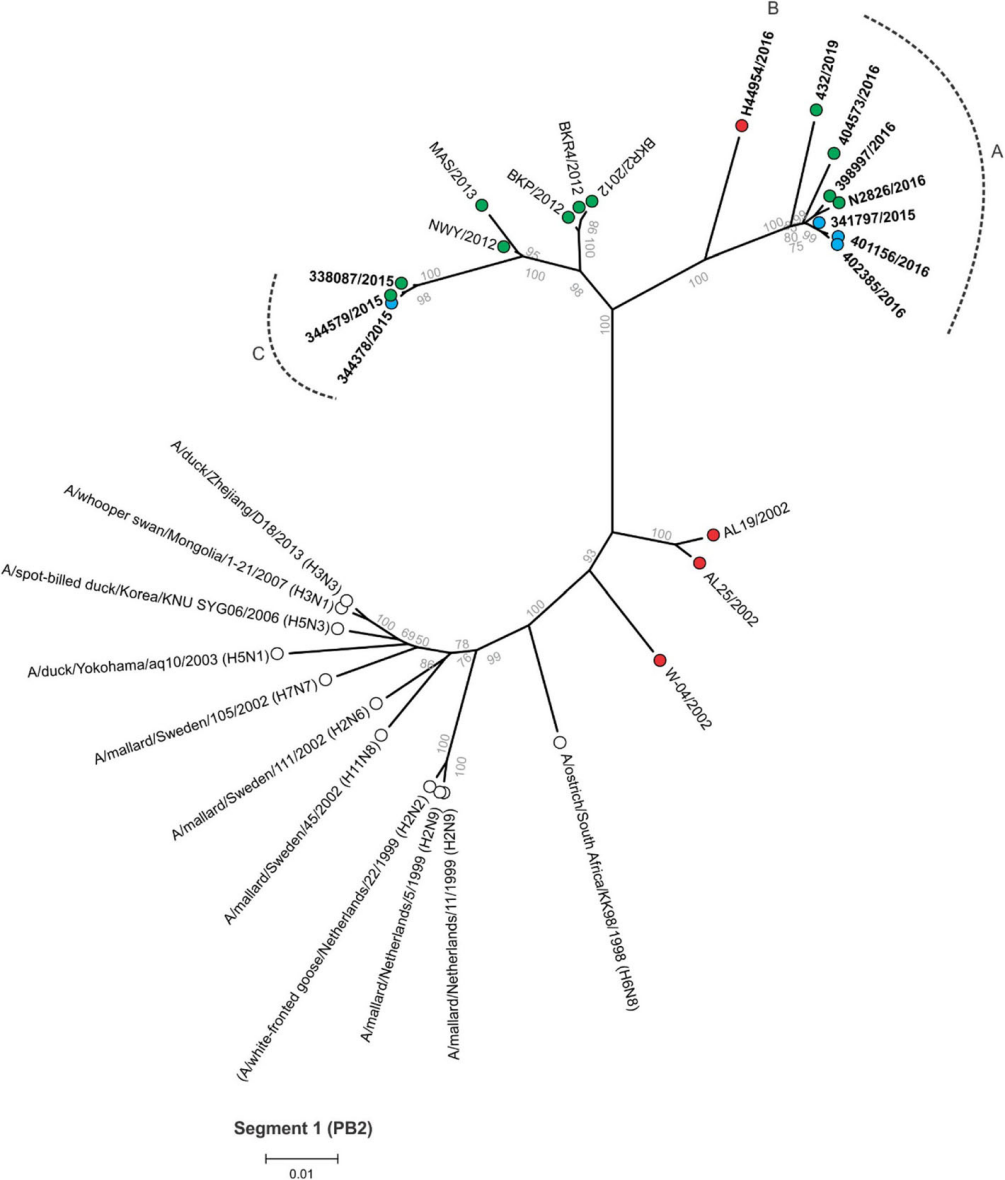
Maximum likelihood phylogenetic tree of segment 1 encoding the polymerase B2 (PB2) protein of South African H6N2 chicken and reference viruses. Green = Gauteng Province; Blue = North West Province; Red = KwaZulu-Natal Province; clear = reference strains. Viruses sequenced in the present study are in boldface. Genetic clusters are labelled A, B and C

**Fig. 5:**
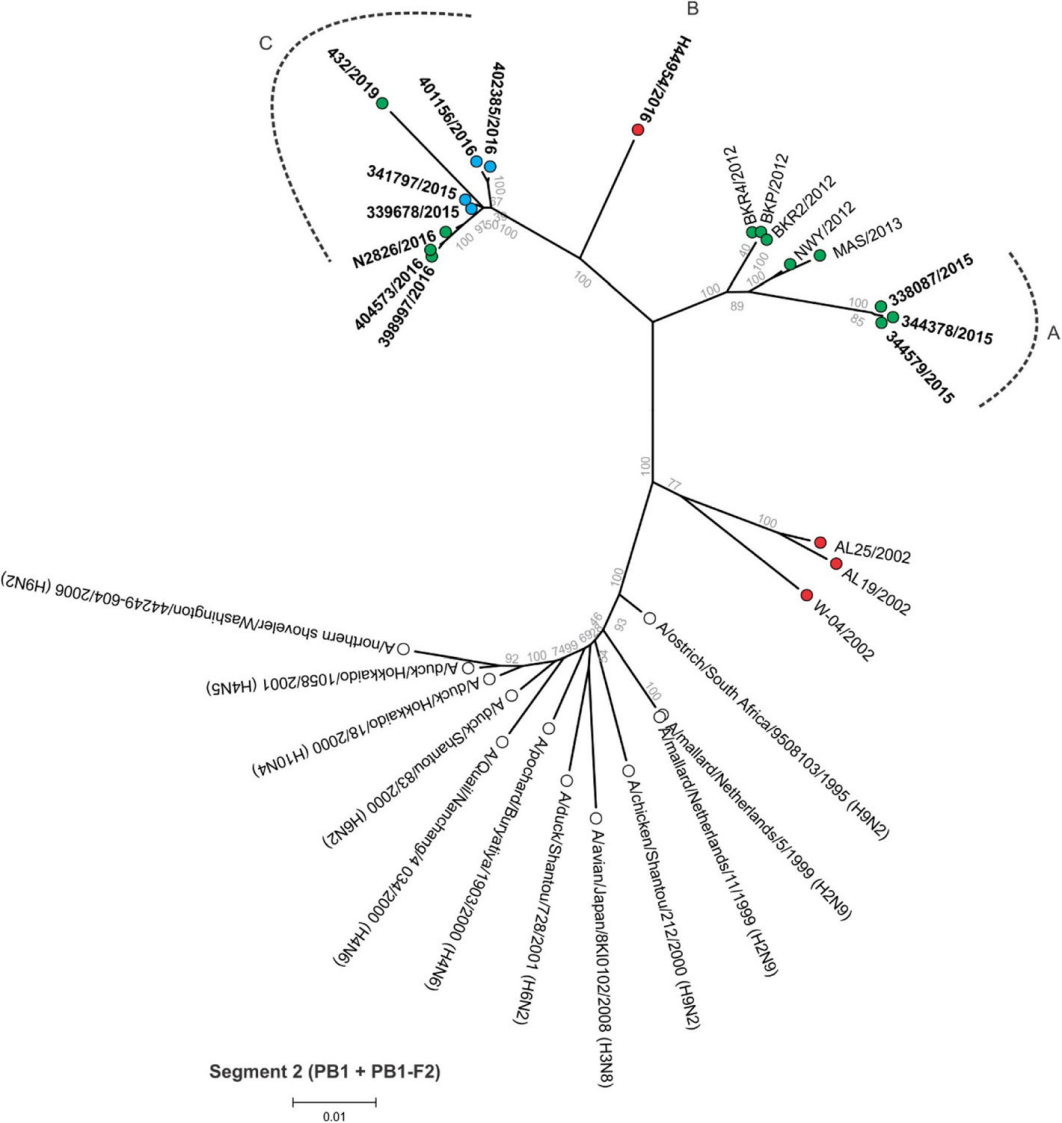
Maximum likelihood phylogenetic tree of segment 2 encoding the polymerase B1 (PB1) and PB1-F2 proteins of South African H6N2 chicken and reference viruses. Green = Gauteng Province; Blue = North West Province; Red = KwaZulu-Natal Province; clear = reference strains. Viruses sequenced in the present study are in boldface. Genetic clusters are labelled A, B and C

**Fig. 6:**
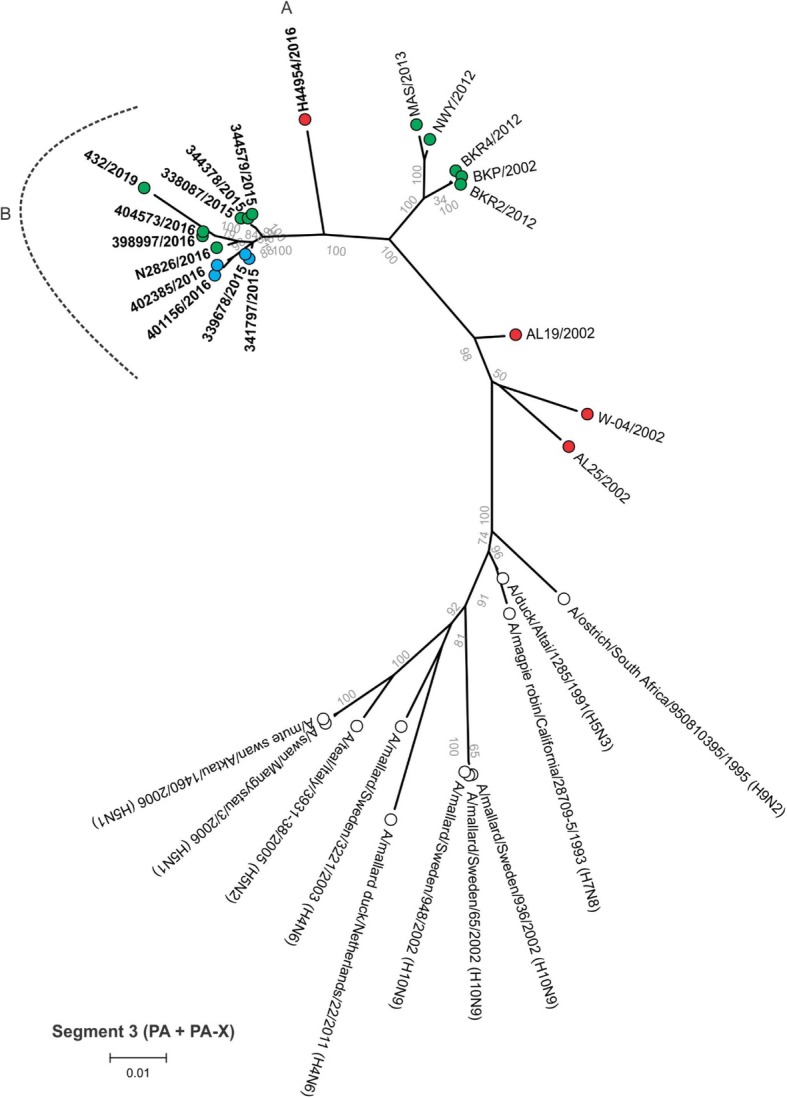
Maximum likelihood phylogenetic tree of segment 3 encoding the polymerase A (PA) and PA-X proteins of South African H6N2 chicken and reference viruses. Green = Gauteng Province; Blue = North West Province; Red = KwaZulu-Natal Province; clear = reference strains. Viruses sequenced in the present study are in boldface. Genetic clusters are labelled A and B and

**Fig. 7:**
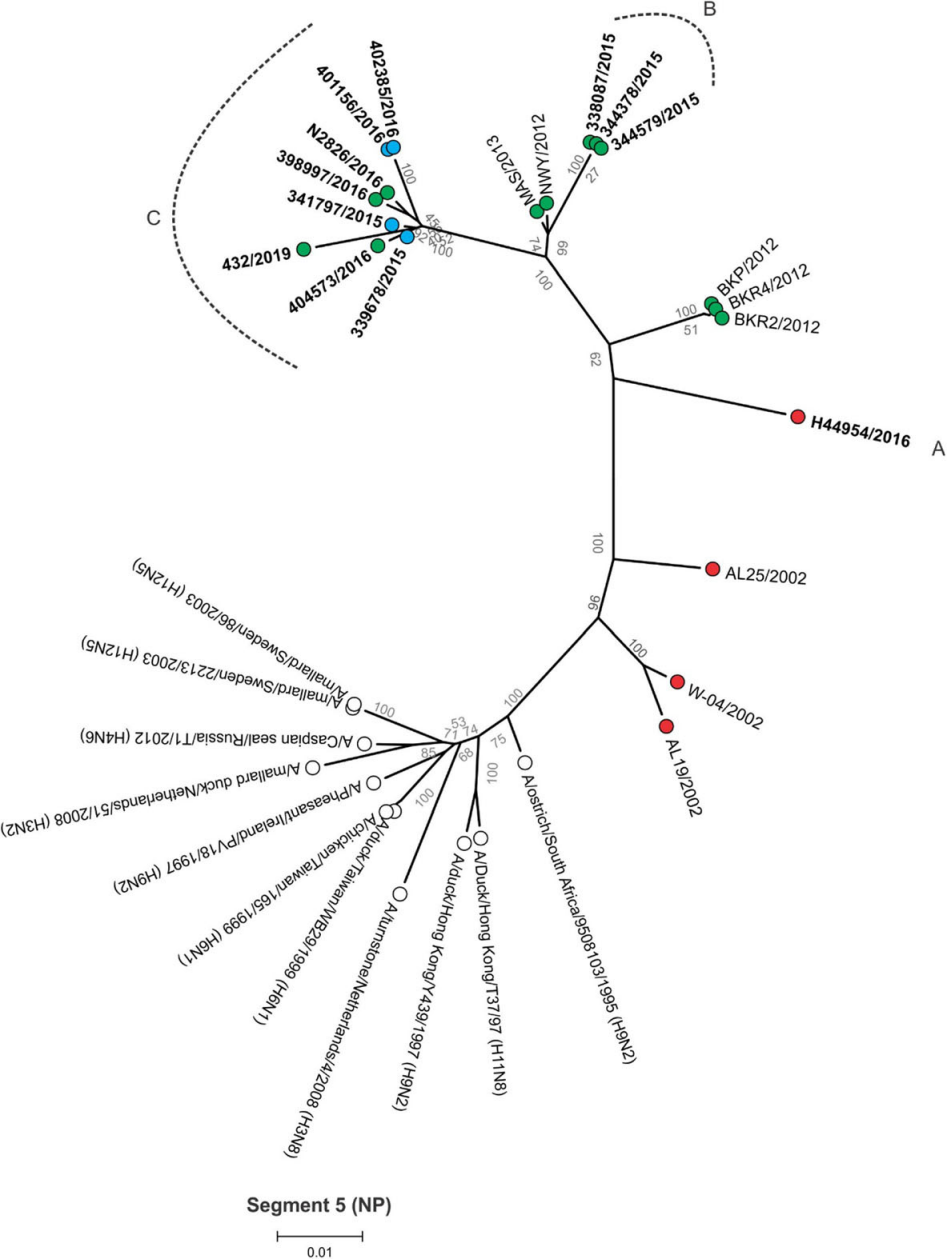
Maximum likelihood phylogenetic tree of segment 5 encoding the nucleocapsid (NP) protein of South African H6N2 chicken and reference viruses. Green = Gauteng Province; Blue = North West Province; Red = KwaZulu-Natal Province; clear = reference strains. Viruses sequenced in the present study are in boldface. Genotypes are labelled A, B and C

**Fig. 8:**
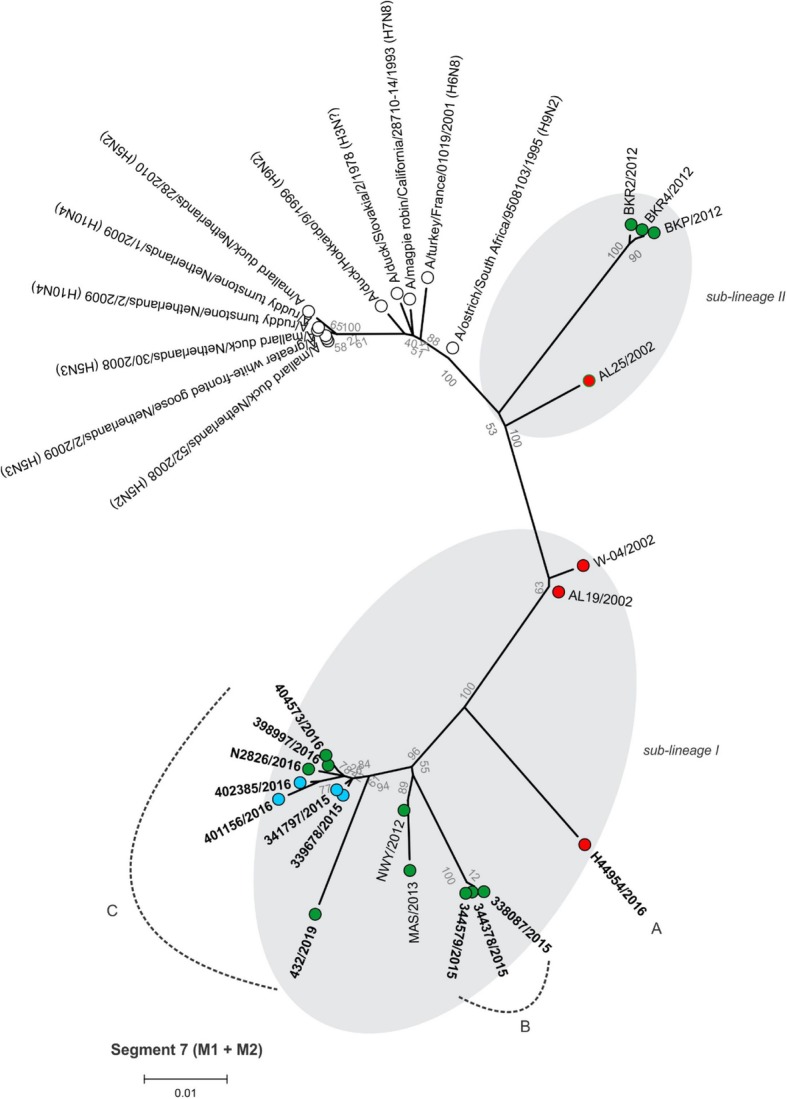
Maximum likelihood phylogenetic tree of segment 7 encoding the matrix proteins 1 (M1) and 2 (M2) of South African H6N2 chicken and reference viruses. Green = Gauteng Province; Blue = North West Province; Red = KwaZulu-Natal Province; clear = reference strains. Viruses sequenced in the present study are in boldface. Genotypes are labelled A, B and C

**Fig. 9:**
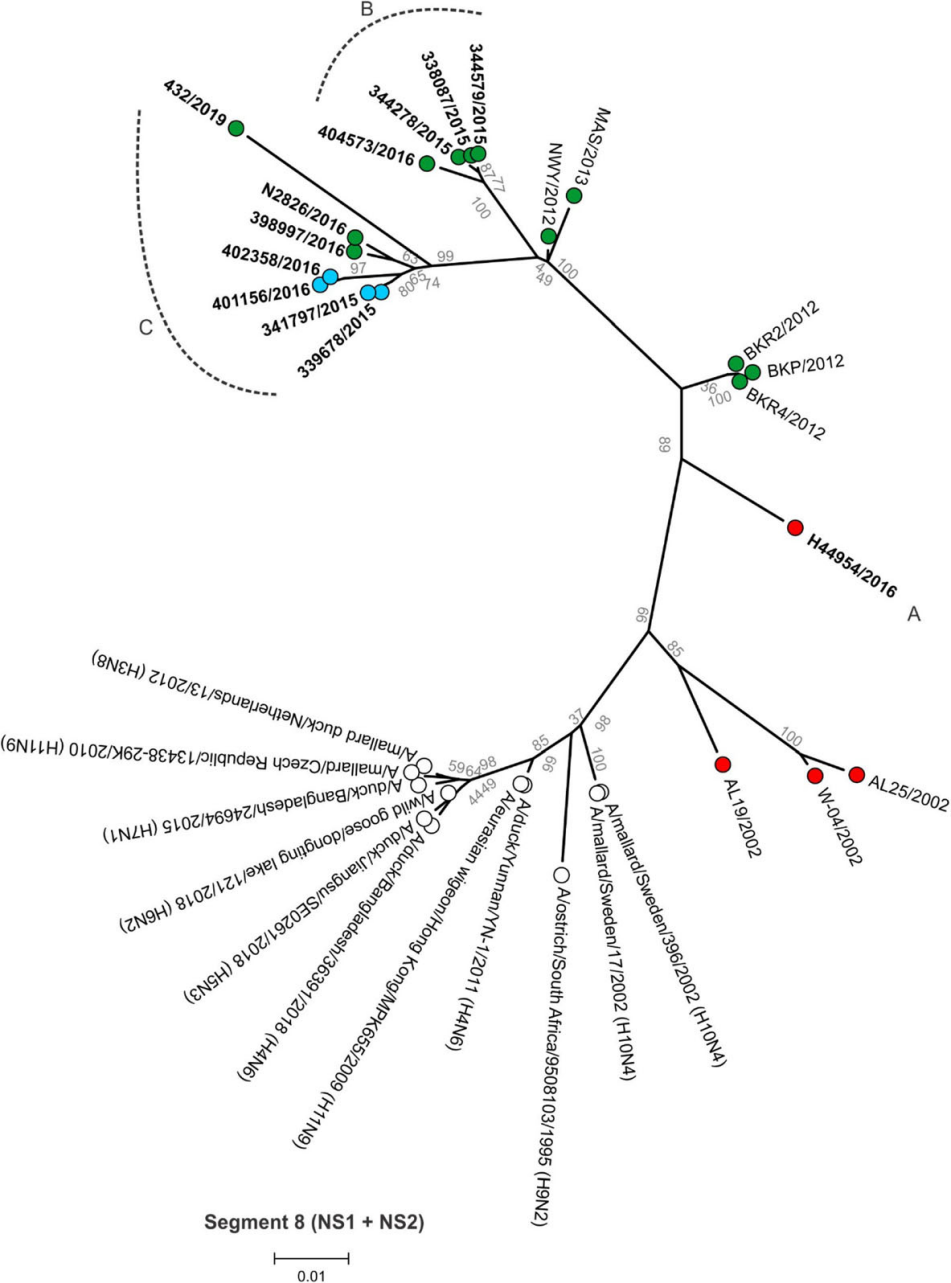
Maximum likelihood phylogenetic tree of segment 8 encoding nonstructural proteins 1 (NS1) and nuclear export proteins (NEP or NS2) of South African H6N2 chicken and reference viruses. Green = Gauteng Province; Blue = North West Province; Red = KwaZulu-Natal Province; clear = reference strains. Viruses sequenced in the present study are in boldface. Genotypes are labelled A, B and C

Between 4.9 and 6.2 million Ion Torrent reads with an average length of 177 base pairs (bp) were generated for each of the 12 sequencing libraries. Maximum likelihood phylogenetic trees (Figs. 2, 3, 4, 5, 6, 7, 8 and 9) were reconstructed for each assembled genome segment to determine the evolutionary relationships with H6N2 viruses isolated from 2002 to 2013 and closest reference sequences retrieved from the Genbank sequence database [22, 23]. Genetic clusters A to C were assigned based on topology to evaluate genome reassortment events.

Separation into sub-lineages I and II was evident in the phylogenetic trees for the surface glycoproteins encoded by segment 4 (HA, Fig. 2) and segment 6 (NA, Fig. 3). Each sub-lineage contained the early isolates from 2002 at the root. Progenitor strains A/ostrich/South Africa/KK98/1998 (H6N8) and A/ostrich/South Africa/9508103/1995 (H9N2) were more closely rooted to sub-lineage II in Figs. 2 and 3, respectively. None of the viruses isolated from 2015 onward were classified as sub-lineage II.

In segment 1 (PB2 gene; Fig. 4), segment 2 (PB1 and PB1-F2 genes, Fig. 5), segment 3 (PA and PA-X genes, Fig. 6), segment 5 (NP gene, Fig. 7) and segment 8 (NS1 and NEP genes, Fig. 9), no separation into sub-lineages I and II was observed, instead grouping was chronological with the late viruses clustering separately from the early strains. Segment 7 that encodes the M1 and M2 genes was the exception where strains separated into sub-lineages I and II as with HA and NA (Fig. 8).

Genome reassortments were assessed by arranging the assigned genetic clusters in Table 2, where the combination of eight segments formed the genotype. Isolates 338,087/2015, 344,378/2015 and 344,579/2015 that cluster closely together in all eight phylogenetic trees (Figs. 2, 3, 4, 5, 6, 7, 8 and 9) shared the same genotype (a). The latter two are from the same site. Both locations were in the Gauteng province, but 338,087/2015 was isolated from broilers whereas the source of 344,378/2015 and 344,579/2015 was a layer farm (Table 1). Isolates 341,797/2015, 339,678/2015, 398,997/2016, N2826/2016, 401,156/2016 and 402,385/2016 also shared the same genotype (b). The first two viruses were isolated from the same site in the North West province. Apart from 402,385/2016 and 401,156/2016, also from the North West Province but isolated a year after 341,797/2015 and 339,678/2015, the other viruses in this genotype originated from the Gauteng province. Strains 404,573/2016 and 398,997/2016 were isolated from the same site, 1 month apart. Reassortment in segments 6 and 8 of 404,573/2016 (genotype c) indicated that this was not a single outbreak, but instead the farm experienced a re-introduction of H6N2. Strain H44954/2016 from KwaZulu-Natal was distinctive; its genotype (d) and position in the phylogenetic trees suggests that the KwaZulu-Natal Province might have variants that are antigenically distinct from the Gauteng and North West Provinces. The most recent isolate, 432/2019 (Gauteng Province) also had a unique genotype (e), its long branches indicated progressive genetic drift from the 2015/2016 isolates, but it shared recent common ancestors in all segments with the Gauteng and North West Province strains apart from segment 4 (HA). Here the HA gene was more closely related to that of H44954/2016 from the KwaZulu-Natal Province (Fig. 2).

**Table 2:** Reassortment analysis of H6N2 virus genomes based on phylogenetic clusters A, B, and C

Strain	Genome segment								Genotype
	1	2	3	4	5	6	7	8	
338,087/2015 (GAU, Br)	C	A	B	B	B	B	B	B	***a***
341,797/2015^a^ (NW, Br.B)	A	C	B	C	C	C	C	C	***b***
339,678/2015^a^ (NW, Br.B)	A	C	B	C	C	C	C	C	***b***
344,378/2015^b^ (GAU, L)	C	A	B	B	B	B	B	B	***a***
344,579/2015^b^ (GAU, L)	C	A	B	B	B	B	B	B	***a***
398,997/2016^c^ (GAU, L)	A	C	B	C	C	C	C	C	***b***
N2826/2016 (GAU, Br)	A	C	B	C	C	C	C	C	***b***
401,156/2016 (NW, L)	A	C	B	C	C	C	C	C	***b***
402,385/2016 (NW, Br.B)	A	C	B	C	C	C	C	C	***b***
404,573/2016^†^ (GAU, L)	A	C	B	C	C	B	C	B	***c***
H44954/2016 (KZN, L)	B	B	A	A	A	A	A	A	***d***
432/19/2019 (GAU, L)	C	C	B	A	C	C	C	C	***e***

*GAU* Gauteng Province, *NW* North West Province, *KZN* KwaZulu-Natal Province, *Br* broilers, *Br.B* broiler breeders, *L* layers (see Table 1). Lower case  bold italicised letters in the last column summarise shared genotypes

^abc^Strains marked with the same symbol are from the same producer/site

### Genetic drift and antigenic diversity in the HA protein

At the nucleotide sequence level in the HA gene, identities between the H6N2 viruses isolated in 2015/2016 ranged from 99.9 to 93.8%, but the similarity of the 2015/2016 viruses with the single virus isolated in 2019 ranged from 96.6% to as low as 92.6% (Additional file 1: Table S1a). When translated to amino acids which reflect the antigenic variation, identities between 2015/2016 viruses ranged from 100 to 94.8%, whereas the 2015/2016 viruses had a range of 96.7 to 93.8% sequence identity with the single virus from 2019 (Additional file 2: Table S1b).

To further evaluate variation at the amino acid/antigenic level over time, all viruses in sub-lineage I were grouped according to year of isolation, and the distances between groups were calculated in MEGA 5 (Additional file 3: Table S1c). Between the 2002 virus group and the 2012/2013 virus group homology was 96.1%, between the 2002 virus group and the 2015/2016 virus group homology was 95.6% and between the 2002 virus group and the single 2019 virus homology was only 90.5%. Between the 2012/2013 virus group and the 2015/2016 virus group the homology was 96.7%, and over the subsequent 3-year period the sequence homology between the 2012/2013 group and the single 2019 virus had dropped to 95.7%. Amino acid sequence homology between the 2015/2016 group and the 2019 virus was 94.7%.

### Molecular markers of host adaptation and virulence across the genome

The HA receptor binding site is surrounded by antigenic sites that are recognised by the most potent neutralizing antibodies. The linear motif RYVRMGTESMN (residues 199 to 205, Additional file 4: Figure S1, H3 numbering), located on the surface on the globular head of the H6 HA protein, was identified as a highly conserved H6 neutralizing B-cell epitope in monoclonal antibody-binding studies [25]. All sub-lineage I viruses isolated from 2015 onwards contained a V201I mutation in this epitope, and 338,087/2015, 344,378/2015 and 344,579/2015 had a M204 L mutation.

Key mutations near the receptor binding site in the HA protein implicated in the switch between H6 viruses binding avian-type receptors to human-type receptors include A138S, I55V, P186L, V187D, E190V, A193N, G225D, Q226L and G228S (H3 numbering [26–28];). Of the aforementioned, the A138S mutation was absent in the H6 ostrich progenitor but appeared in South African H6N2 sub-lineage I viruses as early as 2002, and have been maintained up until 2019, with the exception of 401,156/2016 and 402,385/2015 in which an 138 L mutation was present (Additional file 4: Figure S1). The V187D mutation was already present in the H6N8 ostrich progenitor virus and has persisted through both sub-lineages I and II to the present time. The A193N mutation was present in all sub-lineage I strains, but absent in the progenitor and sub-lineage II viruses apart from AL25/2002. The remaining six mutations in the H6 HA, I155V, P186L, E190V, G225D, Q226L and G228S were not detected.

The HA_0_ motif of PQVETRGIF was present in all viruses sequenced here, except for H44954/2016 that acquired an additional E343G mutation in its motif of PQVGTRGIF (Additional file 4: Figure S1).

N-glycosylation patterns in the HA across all viruses isolated since 2015 were conserved, apart from N2826/2016 that lost N-glycosylation sites at residues 11 and 298 (H3 numbering, Additional file 5: Table S2, Additional file 4: Figure S1). Two predicted O-glycosylation sites were uniform across isolates apart from N2826/2016 that lacked the predicted site at K314.

Like the HA, the NA glycoprotein consists of a globular head and a stalk region. Early studies identified seven antigenic sites on the N2 NA globular head region [29]. Of these, the R344K mutation first emerged in sub-lineage I viruses in 2012/2013 (Additional file 6: Figure S2) and was also found in four of the 12 viruses from 2015 onwards, namely 338,087/2015, 344,579/2015, 390,997/2016 and 404,573/2016.

Most isolates from 2015 onward contained seven predicted N-glycosylation sites across the NA protein (Additional file 5: Table S2; Additional file 4: Figure S2). As with the HA protein, N2826/2016 was the exception, lacking four sites at residues 200, 309, 313 and 402 (full-length N2 numbering). Five other viruses, 339,678/2015, 341,797/2015, 398,997/2016, 401,156/2016 and 402,385/2016 also lacked a single the N-glycosylated site at position 309. These five viruses plus 432/2019 also lacked the predicted O-glycosylation site at 351. Isolate 432/2019 lacked the predicted O-glycosylated site at residue 334 that was common to all others, but all viruses contained the predicted O-glycosylation site at 145. H44954/2016 contained two additional O-glycosylation sites at residues 332 and 336.

The neuraminic acid binding site of NA is situated in the globular head, and the residues responsible for the catalytic function are constant for all NA subtypes in IAVs. However, in response to mutations in the HA that decrease its receptor-binding affinity (for example glycosylation), NA is also capable of evolving a second sialic acid binding site, called the HB site, that functionally replaces the HA binding site [30, 31]. The HB site is characterised by three loops. One loop is formed by a serine triplet, S367, S370 and S372, a second loop formed from residues 400 to 403 by chain interactions with W403, and K432 is involved in the formation of the third loop from 430 to 433 (Additional file 6: Figure S2). The serine triplet is present in a conserved region of the N2 sequences of all the isolates we studied. The tryptophan of the second loop is also present in all viruses, but adjacent to this an N-glycosylation site is present in all viruses except for N2826/2016. In the third loop of the HBS, all sub-lineage I strains (but not sub-lineage II) except for H44934/2016 contain a Q432R mutation. Human influenza viruses usually lack the HB site amino acid sequence [30].

The NA stalk region of sub-lineage I H6N2 viruses is characterised by a 21 amino acid deletion [22, 23] which was present in all the viruses analysed here (Additional file 6: Figure S2), but an additional single codon (serine) was deleted at residue 46 in isolate 432/2019.

The NA protein is a target for antiviral therapy. Six mutations are associated with the acquisition of resistance to neuramindase inhibitors, namely E119V, R152K, D198N, H274Y, R292K and N294S [32]. V96A is specifically associated with resistance to zanamivir and oseltamivir [33], however none of these mutations were present in the neuraminidase proteins of the South African H6N2 viruses (Additional file 6: Figure S2).

In the PB2 protein, K73R, A199S, L475 M, D567N, E627K, A661T and K702R mutations are associated with IAVs capacity to infect humans [34–36]. All were absent from the South African H6N2 viruses apart from K702R (Additional file 7: Figure S3). The K702R mutation was present in the H6N8 progenitor virus and in one early sub-lineage II isolate AL25/2002 but was absent from the later three sub-lineage II strains in 2012. Similarly, the mutation was present in the early sub-lineage I viruses AL19/2002 and W-04/2002, but absent from the two strains isolated in 2012/2013. It however was present in all the sub-lineage I viruses post 2015 apart from three Gauteng isolates: 338087/2015, 344,378/2015 and 344,579/2015. D701N in the PB2 protein was associated with high pathogenicity in mice [37], this mutation was present in early sub-lineage I strain AL19/2002 but was not detected in any other South African H6N2 strains.

H99Y and I368V mutations in the PB1 protein are associated with airborne transmission among ferrets [38] but were absent from the H6N2 viruses. Neither of the human-associated markers in the PB1-F2 protein, R79Q and L82S [35] were detected, nor was N66S that is associated with increased virulence, replication efficiency and antiviral response in mice. No human-associated markers in the PA protein, namely P28L, D55N, V100A, K356R, Q/T/S400 L or T552S [34–36] were detected (data not shown). Human-associated markers in the NP protein (V33I, I109V), M2 protein (L55F) NS1 protein (E227R/K) and NS2 protein (S70G) [34–36] were similarly absent from the H6N2 viruses (data not shown).

### Comparison of assays and antigens used for serology tests

No clinical signs were observed in any of the Specific Pathogen Free (SPF) chickens throughout the animal experiment. A serial titration experiment with H6N2 antisera was used to demonstrate the relative detection limits of the approved methods used for H6 antibody testing in South Africa, especially where specific antibodies in a serum sample are low. In Table 3, columns represent groups of chickens that were either vaccinated with AVIVAC® AI whole virus inactivated H6N2 vaccine (A and B) or not (C), not exposed to field challenge (A) or challenged with H44954/2016 (B and C). Undiluted sera and sera titrated up to 1:256 were tested with two ELISA tests and three HI antigens. The BioChek ELISA detected IAV-specific antibodies in only the undiluted serum of the chicken that was vaccinated with AVIVAC® AI but not exposed to field virus (group A). IDEXX ELISA results were negative. H6N2-specific antibodies were detected by HI up to the 1:8 titration using the 2002 H6N2 antigen (homologous with the AVIVAC® vaccine) and the 2016 H6N2 antigen (homologous with the field challenge virus), but HI using the H6N8 antigen only detected H6N2 antibodies at the positive threshold cutoff of 4 log_2_.

**Table 3:** Detection of H6N2 antibodies in three field scenarios using hemagglutination inhibition (HI) assays and ELISAs

	A Vaccinated chicken, no field challenge*					B Vaccinated chicken with field challenge**					C Non-vaccinated chicken with field challenge***				
Serum titration	ELISA		HI log_2_ titre			ELISA		HI log_2_ titre			ELISA		HI log_2_ titre		
	BioChek titre^1^	IDEXX S/N^2^	2002 H6N2_Ag_	1998 H6N8_Ag_	2016H6N2_Ag_	BioChek titre^1^	IDEXX S/N^2^	2002 H6N2_Ag_	1998 H6N8_Ag_	2016 H6N2_Ag_	BioChek titre^1^	IDEXX S/N^2^	2002 H6N2_Ag_	1998 H6N8_Ag_	2016 H6N2_Ag_
0	**667 (1)**	0.88	**8**	**4**	**9**	**10,755(7)**	**0.15**	**12**	**8**	**12**	**5753 (4)**	**0.23**	**8**	**5**	**12**
1:1	336 (0)	0.56	**7**	2	**7**	**7383 (5)**	**0.13**	**10**	**7**	**11**	**3910 (3)**	**0.12**	**5**	2	**10**
1:2	124 (0)	0.72	**6**	2	**6**	**4850 (4)**	**0.49**	**9**	**7**	**10**	**2296 (2)**	**0.20**	**4**	2	**9**
1:4	50 (0)	0.71	**5**	1	**5**	**3802 (3)**	**0.44**	**8**	**6**	**10**	**1494 (2)**	**0.13**	3	2	**8**
1:8	1 (0)	0.74	**4**	0	**4**	**2155 (2)**	**0.17**	**7**	**5**	**8**	**869 (1)**	**0.23**	2	0	**7**
1:16	1 (0)	0.79	3	0	3	**1107 (1)**	**0.21**	**6**	**4**	**6**	556 (0)	**0.34**	2	0	**6**
1:32	1 (0)	0.83	3	0	3	556 (0)	**0.27**	**5**	2	**4**	331 (0)	**0.28**	1	0	**5**
1:64	1 (0)	0.73	2	0	2	253 (0)	**0.48**	**4**	2	3	131 (0)	**0.40**	0	0	**4**
1:128	1 (0)	1.1	1	0	1	96 (0)	0.66	3	1	3	82 (0)	0.56	0	0	3
1:256	1 (0)	1.07	0	0	1	12 (0)	0.87	2	0	3	1 (0)	0.65	0	0	3

* Prime-boost vaccinated with commercial inactivated whole virus H6N2 vaccine (strain W-04/2002)

** Vaccinated once with commercial H6N2 inactivated vaccine (strain W-04/2002), challenged with H6N2 strain H44954/2016

*** Challenged with H6N2 strain H44954/2016

^1^Titer group in brackets

^2^Sample to negative ratio

Ag-Antigen

Positive serological test results are highlighted in boldface

In the vaccinated chicken exposed to field virus (group B), both ELISAs detected influenza virus-specific antibodies, BioChek assay up to the 1:16 titration and IDEXX assay up to the 1:64 titration. The 2002 H6N2 antigen was the most sensitive on HI, detecting serotype-specific antibodies up to the 1:64 titration, with the H6N8 antigen detecting antibodies up to the 1:16 titration and the 2016 H6N2 antigen faring slightly better at the 1:32 titration.

The non-vaccinated chicken exposed to the field virus elicited a humoral response that was strongly detectable by the IDEXX ELISA (up to 1:64) and less robustly by the BioChek assay (up to 1:8). Whereas the 2002 H6N2 antigen and the 1998 H6N8 antigen only detected serotype-specific antibodies up to the 1:2 titration and undiluted sera, respectively, the 2016 H6N2 antigen detected H6N2-specific antibodies up to the 1:64 dilution.

## Discussion

### H6N2 reassortment, antigenic diversity and evolution

The function of the HA glycoprotein is to attach to host cell receptors to initiate infection and the complimentary role of the NA glycoprotein is to complete the infectious cycle by cleaving terminal sialic acids on host cells and the newly formed virions to facilitate release and dispersal*.* HA is immunodominant, but NA-specific antibodies are also strongly neutralizing [31].

The antigenic separation into sub-lineages I and II was evident in the phylogenetic analyses of the HA and NA proteins but evolutionary patterns were markedly different in the segments encoding the internal non-structural proteins. Segment 7 (encoding the M1 and M2 proteins) was the exception, where like the surface glycoproteins it separated into sub-lineages I and II. Although this phenomenon was noted previously [23], the reason for this is still not understood. The M1 protein interacts with the HA and NA glycoproteins during assembly of the virus particle [39], therefore the co-assortment of these three segments might reflect specific structural complementation in the proteins they encode, but further investigations are required for clarity.

For the first time, sufficient numbers of isolates were available to assess reassortment patterns in local H6N2 strains. A specific genotype was isolated from broiler breeders, broilers and layer birds, indicating epidemiological connections not only between provinces, but also between different farming systems. Reassortment patterns also revealed that inter-provincial mixing had occurred with viruses originating in the Kwa-Zulu Natal province. Notably, only South African lineage chicken H6N2 gene segments were detected, i.e. there was no reassortment with wild bird or other ostrich IAVs.

Even though an accurate estimation of antigenic drift was hindered by relatively low numbers of viruses available for analysis, distance matrices of nucleotide and amino acid sequence identities were used to approximate the extent of antigenic variation present in the field. Between 2002 and 2013 (an 11-year period) amino acid sequence homology dropped to 96.1% (i.e. a 3.9% decrease over 10 years) but from 2015/2015 to 2019 the homology was only 94.7% (i.e. a 5.3% decrease over 3 years). It is therefore likely that our estimates are a gross underestimation of the actual variation in the field, especially since so few isolates from KZN and no isolates from the Western Cape or other provinces were available for comparison.

The surface glycoproteins HA and NA are constantly exposed to the host immune system and Rauff et al. [23] previously reported that sub-lineage I mutates at the higher rate of 7.7 × 10^− 3^ nucleotide substitutions per site per year compared to 4.05 × 10^− 3^ for sub-lineage II, attributed to vaccine pressure. Amino acid substitution in HA and NA antigenic regions is a well-known modification for escaping host humoral immunity but virus protein glycosylation within the host’s secretory pathway is one of the most abundant and diverse forms of modification, as glycosylation of the HA is essential for protein folding and transport to the cell surface as well as biological functions. Oligosaccharides near the receptor-binding site of HA can alter its binding avidity and specificity for the host’s sialic acid cell surface receptor receptors [40]. The HA and NA glycoproteins work in concert to attach and release viruses from the cell surface and NA stalk length determines the height of the globular domain and therefore its access to substrates and its interactions with HA. A shortened NA stalk is frequently observed during adaptation of IAV between avian species, and is compensated by increased glycosylation in the protein [31, 37] Compared to sub-lineage II viruses, all sub-lineage I strains possessed an NA-stalk deletion and compensatory hyper-glycosylated HA head [22, 23]. Our analyses of N and O-type glycosylation showed variations across time in the number of predicted glycosylated sites in both HA and NA. Most viruses isolated since 2015 had 9 predicted N-glycosylated sites and 2 O-glycosylated sites in the HA but 6 to 7 and 2 to 3 predicted N-glycosylated and O-glycosylated sites in the NA, respectively. H44954/2018 from the KZN province was one of the exceptions with 8 predicted N-glycosylated sites and 5 predicted O-glycosylated sites in the NA protein. Isolate 432/19 had an additional single codon deletion at position 46 in the NA stalk and an additional compensatory predicted N-glycosylation site in the HA compared to all the other sub-lineage I viruses.

The HA_0_ cleavage site motif is a critical determinant of virulence. LPAI viruses including the H6 subtype lack a dibasic amino acid pair that makes HA_0_ cleavable by the subtilisin-like proteases present in wide range of tissues, to trigger lethal systemic infection. HA_0_ motifs of LPAI viruses are instead recognised by trypsin-like enzymes restricted to epithelial cells lining the respiratory and gastrointestinal tracts [41]. The unusual HA_0_ motif of PQVETRGIF (the typical H6 LPAI HA_0_ sequence is PQRETRGLF) in sub-lineage I viruses was evident from 2012 onwards (Additional file 4: Figure S1) but H44954/2016 acquired an additional E343G mutation that produced the unique motif of PQVGTRGIF. The atypical HA_0_ motifs may subtly alter the subset of proteases to enhance infection of chicken respiratory epithelial cells, but this remains to be verified experimentally.

### Molecular markers for human adaptation

The potential zoonotic threat of H6 viruses makes it vital to monitor the molecular markers in protein sequences that are known to switch the affinity from avian-type receptors (α-2,3 linked sialic acids) to human-type receptors (α-2,6 linked sialic acids) and other virulence or antiviral resistance traits. Sub-lineage I H6N2 viruses acquired three of the nine HA mutations associated with human receptor-binding preference (A13S, V187D and A193N) since 2002. Most sub-lineage I viruses isolated since 2015 also acquired the K702R mutation in PB2 associated with the ability to infect humans, whereas prior to 2015 most viruses in sub-lineages I and II contained the typical avian lysine marker.

### The challenges of current serological test methods

Serological testing is the official method used for H6N2 screening in South African poultry. Influenza virions comprise four major structural proteins, viz. M1, HA, NP and NA, present at respective molar ratios of 100, 26, 22 and 3. Under experimental settings, immunization with inactivated whole viruses result in predominant antibody responses to HA (55%), NA (35%) and NP (10%) [42, 43].

Testing is performed in two phases. First, sera are screened for the presence of IAV group antibodies by ELISA; for chickens the indirect BioChek Influenza A ELISA is an example, where the coating antigen is an unspecified whole virus (Luuk Stoker, personal communication). The IDEXX Influenza A inhibition ELISA is more commonly used for non-gallinaceous birds such as commercial ostriches, and this test specifically detects antibodies against the IAV nucleocapsid protein (NP).

The second testing phase entails detection of specific H5, H6 and H7 antibodies. To distinguish steric nonspecific inhibition in the HI assay, it is common practise to use two antigens per serotype, where the antigens have heterologous N-types, and these antigens should be closely- related to the circulating field virus [2]. The current official antigen pair used in South Africa for the H6 subtype is strain W-04/2002 (H6N2), homologous with the AVIVAC® AI inactivated vaccine [23], and ostrich virus KK98/1998 (H6N8). We included H44954/2016, the homologous antigen used for challenge, for experimental purposes (Table 3).

Chickens with vaccine responses only (column A, Table 3) may have negative or low ELISA positive results, but strong H6 antibody specific responses on HI tests (> 8 Log_2_ with H6N2 antigens). Even though a positive ELISA titre was only detected in the undiluted serum with the BioChek ELISA here, inactivated whole-virus vaccines can induce anti-NP antibody responses of up to 10 log_2_ [44]. This makes it impossible to use the ELISA tests to reliably distinguish whole-virus vaccine from field challenge antibody responses.

A vaccinated chicken that was exposed to a field challenge (column B, Table 3) had high ELISA titres as well as HI titres up to 12 log_2_ against the H6N2 antigens and up to 8 log_2_ against the H6N8 antigen, and HI titres were positive against all three antigens even up to the 1:16 dilution, which was also the cut-off for a positive BioChek ELISA result.

The non-vaccinated chicken challenged with the 2016 H6N2 strain (column C, Table 3) illustrates a situation that typically confounds the interpretation of diagnostic test results. The example of the 1:8 titration, where the ELISA result is positive but the HI results for the 2002 H6N2 and 1998 H6N8 antigens are negative, might suggest that the flock was exposed to a non-H6 subtype IAV. In at least one case in 2018 a farm was quarantined by a State veterinarian who misinterpreted the lack of a reaction with the 2002 H6N2 and 1998 H6N8 antigens and the non-specific cross reaction with an H5N2 antigen as an H5 infection. It is however clear from the HI result with the 2016 H6N2 antigen that the sample is actually strongly H6 positive. The protein sequence identity between the 2002 H6N2 antigen and the 2016 challenge virus is only 90.9% (Additional file 2: Table S1b). The mismatch between the 2002 test antigen and circulating field strains has increased steadily over the years, and currently stands at 90.5% identity with strain 432/19. The antigenic mismatch of the 2002 H6N2 antigen with sub-lineage II, if it is still circulating, could therefore be even greater.

## Conclusion

Opinions vary in the local industry on the importance the endemic H6N2 infection. Some veterinarians continue to use the inactivated vaccine because it limits production losses; others consider H6N2 to be of negligible importance. The national veterinary directorate strictly regulates diagnostic reagents (e.g. standardized reference antigens used for HI) and the use of the vaccine, but decentralized veterinary services means that responsibility for disease control falls to the provincial governments. Unfortunately, the general attitude towards H6N2 control remains one of apathy and few efforts are made to submit samples for isolation and characterization where H6N2 infections are suspected.

This study showed that genetic drift in H6N2 continues and that antigenic diversity in sub lineage I is increasing and certainly underestimated; that the viruses are gradually gaining markers associated with the ability to infect humans; and that it’s essential that HI antigens be periodically updated so that infections are diagnosed correctly. The sample size, although small, was able to determine for the first time that inter-provincial spread and reassortment is occurring. The molecular epidemiological links identified between different farming systems once again raises questions about how H6N2 is being maintained and spread in the country, with fomites spread between farms by people and vehicles as the likely route.

Although the endemicity of H6N2 in South Africa doesn’t affect the official disease status of the country for trade, it is highly undesirable for several other important reasons:

1. Reduced profits: H6N2 infection does cause production drops and if producers have calculated their annual financial losses and the associated costs of diagnostic tests, vaccines and medication to treat H6N2-linked infections, the figures are not publicly available but probably significant.
2. Increased use of antibiotics: respiratory viruses such as H6N2 predispose chickens to secondary bacterial infections, increasing the prescribed use of antibiotics and subsequently the local levels of antimicrobial resistance in bacteria such as *E. coli* [45] and mycoplasmas [46]. For South Africa to meet its international commitment to reduce antibiotic usage in feed animals, greater efforts must be made to control viral poultry diseases.
3. Biological risk: the H6N2 virus is highly chicken-adapted and should it reassort with another IAV (e.g. a wild duck LPAI H5/H7 IAV), it could contribute genes that would not require any adaptation to replicate to high levels in poultry and spread rapidly. It is well-established that East Asian H6 viruses were a source of internal protein genes in the pandemic HPAI H5N1 virus [47].
4. Zoonotic threat: sub-lineage I strains are gradually gaining mutations associated with acquiring affinity for human receptors. The continuing genetic and antigenic drift in H6N2 viruses will have to be closely monitored so that human health authorities can be alerted in time. South Africa has one of the highest HIV/AIDS positive populations in the world, and agricultural workers were estimated to have an infection rate double that of the national average [48].

No data exists on whether the H6N2 infection has spread to neighbouring Namibia, Botswana, Zimbabwe, Mozambique and Lesotho or further north into Zambia or other countries during the 18-year course of the epidemic in South Africa, through formal or informal trade. Neither the chicken adapted H6N2 lineages nor their genome segments have ever been detected in commercial ostriches or wild birds surveyed, nor have any further transmission of IAVs of any subtype from ostriches to chickens been recorded since the 1990s [49].

Because a reservoir of infection has never been identified, for a long time it was thought that H6N2 infections have a short window period of viral shedding of about a week to 10 days. A recent vaccine-challenge study with strain H44954/2016 [44] however showed that excessively high levels of viruses (> 8.92 log_10_ viral copies/ml) were shed from the oropharynx in the first 4 days of infection and that 36% of non-vaccinated chickens were still shedding virus 3 weeks after exposure. In comparison, > 5.7 million-fold fewer viral particles were shed from the cloaca and cloacal shedding had ceased completely by 2 weeks post infection. The study also demonstrated that an appropriately antigen-matched vaccine can be very effective in reducing both the magnitude and the duration of H6N2 shedding. On the other hand, a mismatched vaccine prolonged viral shedding compared to the non-vaccinated control group.

Finally, the South African H6N2-endemic status of poultry is entirely different from the East Asian situation. East Asian poultry production is characterized by inter-species mingling in live bird markets, frequent contact between domestic poultry and wild aquatic birds, and the resulting co-circulation of a great diversity of IAV subtypes that continuously undergo reassortment [12, 21]. South Africa’s is a less dense and more geographically isolated system, with few live bird markets to facilitate inter-species mixing, geophysical separation of the major ostrich and chicken-producing areas, very little direct contact between wild aquatic birds and chickens; duck farming is minor and turkey farming is absent, however a large and unregulated cull chicken trading industry transports spent chickens within and between provinces. In 2017/2018 Clade 2.3.4.4 HPAI H5N8 was spread to sub-Saharan Africa by migrant wild birds, resulting in spill-overs to many species, including commercial chickens in South Africa. Apart from this event, no IAV other than H6N2 has ever been detected in local chickens [50]. The H6N2 lineage is mutating and reassorting but has remained genetically pure since it emerged more than 18 years ago, providing a unique opportunity to study IAV evolution in the field, over an extended period, in single specie for now.

## Methods

### Sampling, diagnosis and virus isolation

Tracheal swabs, cloacal swabs or tissue samples (tracheas or organ pools) from flocks that showed typical signs of H6N2 infection such as respiratory symptoms or drops in egg production were submitted by veterinarians to diagnostic laboratories for PCR confirmation. The only flock that had been vaccinated was that from which H44954/2016 was isolated (44). RNAs were extracted with the Quick-RNA miniprep kit (Zymo Research, Irvine, USA). The presence of IAV was confirmed using the M-gene targeted real-time reverse transcription (RT) PCR oligonucleotides described by Spackman et al. [51] with Real-Time Ready Mastermix (Roche, Basel, Switzerland) on a Lightcycler 1.5 (Roche). 3 μl of template RNA was combined with 4.3 μl PCR grade water, 2 μl 5x reaction buffer, 0.5 μl of a 20X primer/probe mix and 0.2 μl enzyme mix. Cycling parameters were as described [51] with fluorescence acquisition at the end of the extension phase. Cycle threshold values < 40 were considered positive. The H6 subtype was confirmed using two step conventional RT-PCR. For reverse transcription with a Revert Aid M-MuLV reverse transcriptase kit (Thermo Fischer Scientific, Waltham, USA), 3 μl RNA template was combined with 4.25 μl PCR grade water, 3 μl 5x reaction buffer, 0.5 μl 10 mM dNTPs, 0.5 μl of primer UNI 12 (5′-AGCAAAAGCAGG − 3′) and 0.25 μl RT enzyme. Reactions were incubated at 42 °C for 1 h and 72 °C for 5 mins on a Kyratec Supercycler (Celtic Molecular Diagnostics, Cape Town, South Africa. PCR amplification was performed with a FastStart PCR Mastermix kit (Roche), where 2.5 μl cDNA template was combined with 9.5 μl PCR grade water, 0.25 μl (30 μM) each of oligonucleotide primer pair H6-F/H6-R (H6-F: 5′-AATCTAATCGCYCCTTGGTATGC-3′; H6-R: 5′-ACCAACAGTYCAGCATTGTATGT-3′) and 12.5 μl master mix. Reactions were cycled at 95 °C for 4 mins followed by 35 cycles of 95 °C for 30 s/ 59 °C for 30s/72°C for 30s, and a final extension step of 72 °C for 7 mins. PCR products of 545 base pairs were visualised under UV light following electrophoresis in 1% agarose.

Virus isolation of PCR-positive samples in 9-to 11 day old embryonated chicken eggs from a SPF flock (AviFarms (Pty) Ltd., Pretoria, South Africa) was performed according to the standard procedure [2] at Deltamune (Pty) Ltd. in Pretoria, the Department of Veterinary Tropical Diseases at the University of Pretoria or RCL Foods laboratory in Pietermaritzburg. The presence of Influenza A virus was confirmed in allantoic fluids by hemagglutination inhibition using specific chicken antisera and RT-PCR as described above.

### Ion torrent sequencing

Total RNA was extracted from infected allantoic fluids with TRIzol® (Thermo Fischer Scientific) or Quick-RNA miniprep kit (Zymo Research) and shipped on ice to the Central Analytical Facility at Stellenbosch University. Barcoded cDNA libraries were prepared with Ion Total RNA-Seq v2 and Ion Xpress™ RNA-Seq Barcode Kits (Thermo Fisher Scientific). Libraries were then purified and assessed for yield and fragment size distribution using the High Sensitivity DNA Kit and chips on the BioAnalyser 2100 (Agilent Technologies, Santa Clara, USA) according to the recommended protocol. After dilution to a target concentration of 80pM, the barcoded cDNA libraries were combined in equimolar amounts for sequencing template preparation using the Ion PI™ HiQ™ Chef Kit (Thermo Fisher Scientific). Enriched, template positive ion sphere particles were loaded onto an Ion PI™ (v3) Chip (Thermo Fisher Scientific). Massively parallel sequencing was performed on the Ion Proton™ System according to the manufacturer’s protocol. Flow space calibration and basecaller analysis were performed using standard analysis parameters in the Torrent Suite Version 5.4.0 Software.

### Sequence analysis

Ion torrent reads were imported into CLC Genomics Workbench 5.2.1. References genome sequences KX595237-KX595244 were used as scaffolds for the assembly of consensus sequences for each genome segment, for each virus isolate. Multiple nucleotide sequence alignments (nucleotide as well as the translated proteins) were prepared in BioEdit v7.2.5 [52] with reference genomes retrieved from Genbank (https://www.ncbi.nlm.nih.gov/nuccore). Phylogenies were reconstructed using the Maximum Likelihood statistical method in MEGA v5.5.2 [53], with 1000 bootstrap replicates. The Tamura-Nei nucleotide substitution model was used, specifying a uniform rate among sites. Trees were inferred with a Nearest-Neighbour-Interchange method, with a very strong brand swap filter*.* MEGA v5.5 was also used to generate distance matrices and estimate average evolutionary divergence in the HA protein sequence pairs, expressed as percent sequence identity, within and between groups that were defined according to year of isolation.

The NetNGlyc 1.0 Server (http://www.cbs.dtu.dk/services/NetNGlyc/) was used to predicts N-Glycosylation sites in the HA and NA proteins, using artificial neural networks that examine the sequence context of Asn-Xaa-Ser/Thr sequons. The NetOGlyc 4.0 Server (http://www.cbs.dtu.dk/services/NetOGlyc/) was used for neural network predictions of mucin type GalNAc O-glycosylation sites in the HA and NA proteins [54].

### Production of H6N2 antisera

Two groups (*n* = 10 each) of SPF White Leghorn chickens, both male and female at 5 weeks of age (AviFarms (Pty) Ltd., Pretoria) were assigned randomly and housed in isolators inside the Poultry Biosafety Level 3 facility at the University of Pretoria’s Faculty of Veterinary Science. The room had natural light. Commercial feed and water were provided ad libitum for the duration of the trial. Birds were observed daily for clinical signs. Groups 1 and 2 were vaccinated with AVIVAC® AI H6N2, a whole inactivated oil emulsion vaccine (Deltamune, (Pty) Ltd., Pretoria) that is produced from sub-lineage I seed strain W-04/2002, intra-muscularly in the breast with 0.5 ml of the suspension at day 0 of the trial. Group 3 was not vaccinated. At day 21, all chickens were bled, collecting 1 ml of blood from the wing vein, and only group 1 received a booster vaccination. Two weeks later, at day 35, all chickens were bled again as before, and all were subsequently challenged with a dose 10^6^ EID_50_ of H6N2 strain H44954/2016, also a sub-lineage I strain, via the intranasal and intraocular routes. Blood was collected at days 42 (7 days post challenge) and 49 (14 days post challenge), before euthanasia via cervical dislocation. Sera were left to clot at room temperature for at least 1 h before centrifugation at 5000 X G for 10 mins in an Eppendorf 5804R centrifuge (Hamburg, Germany).

All sera were tested by hemagglutination inhibition (HI) assay at the Serology laboratory, Department of Veterinary Tropical Diseases (see below) and the antiserum with the strongest H6N2-specific titre (> 10 log_2_) at each of three sampling points as follows was selected for the comparative study: an AVIVAC® AI prime-boost vaccinated chicken from group 1 at day 35 (prior to challenge), representing a bird with strong vaccine-induced immunity but not exposed to a field virus; a single AI-vaccinated chicken from group 2 at day 49, representing a vaccinated bird that was exposed to field challenge; and a bird from non-vaccinated group 3 at day 49, representing a naïve bird exposed to a field virus. A two-fold serial titration (up until 1:256) of each of the three H6N2 antisera was prepared in SPF negative chicken serum.

### Serological tests

HI assays were performed according to the internationally standardized procedure [2]. The antigen pair used routinely for detection of H6N2-specific antibodies in poultry in South Africa is W-04/2002 which is the seed strain for the AVIVAC® AI vaccine [23] and an H6N8 antigen, A/ostrich/South Africa/KK98/1998, a progenitor of the South African chicken H6N2 lineages [22]. Both antigens are produced as standardized reference antigens by Deltamune (Pty) Ltd., Pretoria. Strain H44954/2016 was propagated at UP for use as an additional test antigen in this study. HI titres ≥1:16 or 4 log_2_ were considered as positive.

Sera were tested with two commercial ELISAs. BioChek AI ELISA (Reeuwijk, The Netherlands), an indirect chicken-specific test, was performed at the UP Department of Veterinary Tropical Disease according to the manufacturer’s recommended protocol. Antibody titres were calculated from the sample to positive (S/P) ratio in BioChek software that also assigns a titre group. Antibody titres ≥668 (titre group ≥1) are considered positive. IDEXX Influenza A virus Antibody test (Hoofddorp, The Netherlands), a multispecies competition ELISA was used according to the manufacturer’s instructions, with an iMark™ Microplate Reader (BioRad, Hercules, USA). The sample to negative control (S/N) ratio was calculated from the optical density at A^655^ for each sample. An S/N ratio < 0.5 is considered positive.

## Supplementary information

**Additional file 1: Table S1a.** Percentage nucleotide sequence identity in the HA genes of sub-lineage I viruses isolated since 2015.

**Additional file 2: Table S1b.** Percentage amino sequence identity in the HA proteins of all sub-lineage I viruses.

**Additional file 3: Table S1c.** Amino acid between-group distances.

**Additional file 4: Figure S1.** Alignment of the hemagglutinin protein sequences of South African H6N2 isolates from chickens.

**Additional file 5: Table S2.** Predicted glycosylation patterns in the surface glycoproteins of H6N2 influenza viruses isolated since 2015.

**Additional file 6: Figure S2.** Alignment of the neuraminidase protein sequences of South African H6N2 isolates from chickens.

**Additional file 7: Figure S3.** Alignment of the polymerase B2 (PB2) protein sequences of South African H6N2 isolates from chickens.

## Data Availability

The genome sequences supporting the conclusions of this article are available in the GenBank repository under the accession numbers listed in Table 1. The datasets supporting the conclusions of this article are included within the article and its additional files.

## Abbreviations

bp: Base pairs
ELISA: Enzyme-linked immunosorbent assay
GAU: Gauteng Province
H: Hemagglutinin subtype
HA: Hemagglutinin protein
HA0: Hemagglutinin protein cleavage site
HI: Hemagglutination inhibition
HIV/AIDS: Human immunodeficiency virus/ acquired immunodeficiency syndrome
IAV: Influenza A virus
KZN: KwaZulu-Natal province
M1: Matrix protein 1
M2: Matrix protein 2
M-MuLV: Moloney murine leukemia virus
N: Neuraminidase subtype
NA: Neuraminidase protein
NS1: Non-structural protein 1
NS2: Non-structural protein 2
NW: North-West province
OIE: *Office International des Epizooties* /World Organization for Animal Health
PA: Polymerase A protein
PA-X: Polymerase A-X protein
PB1: Polymerase B1 protein
PB1-F2: Polymerase B1-F2 protein
PB2: Polymerase B2 protein
RBC: Red blood cell
rRT-PCR: Real-time reverse transcription polymerase chain reaction
RT-PCR: Reverse transcription polymerase chain reaction
SPF: Specific pathogen free
