# Supplementary material for: Continuing evolution of H6N2 influenza a virus in South African chickens and the implications for diagnosis and control
Source: BMC Vet Res. 2019 Dec 18;15:455. doi: 10.1186/s12917-019-2210-4 (PMC6921544; doi:10.1186/s12917-019-2210-4)
Supplement: Supplementary file 1 — Additional file 1: Table S1a. Percentage nucleotide sequence identity in the HA genes of sub-lineage I viruses isolated since 2015. [file 12917_2019_2210_MOESM1_ESM.docx]

**Table S1a. Percentage nucleotide sequence identity in the HA genes of sub-lineage I viruses isolated since 2015**

| Isolate | **338087/15** | **339678/15** | **341797/15** | **344378/15** | **344579/15** | **398997/16** | **401156/16** | **402385/16** | **404573/16** | **N2826/16** | **H44954/16** |
| --- | --- | --- | --- | --- | --- | --- | --- | --- | --- | --- | --- |
| 338087/15 |  |  |  |  |  |  |  |  |  |  |  |
| 339678/15 | 95.6% |  |  |  |  |  |  |  |  |  |  |
| 341797/15 | 95.6% | 99.9% |  |  |  |  |  |  |  |  |  |
| 344378/15 | 99.6% | 95.9% | 95.9% |  |  |  |  |  |  |  |  |
| 344579/15 | 99.5% | 95.7% | 95.7% | 99.9% |  |  |  |  |  |  |  |
| 398997/16 | 95% | 99.2% | 99.2% | 95.2% | 95.0% |  |  |  |  |  |  |
| 401156/16 | 95.2% | 99.0% | 99.0% | 95.4% | 95.03% | 98.4% |  |  |  |  |  |
| 402385/16 | 95.2% | 99.0% | 99.0% | 95.4% | 95.02% | 98.4% | 99.8% |  |  |  |  |
| 404573/16 | 95.5% | 99.2% | 99.2% | 95.2% | 95.1% | 99.7% | 98.5% | 98.4% |  |  |  |
| N2826/16 | 95.4% | 99.4% | 99.4% | 95.6% | 95.4% | 99.2% | 98.7% | 98.6% | 99.2% |  |  |
| H44954/16 | 94.9% | 94.5% | 94.5% | 95.1% | 95.0% | 93.8% | 94.0% | 93.9% | 93.9% | 94.4% |  |
| 432/19 | 93.2% | 93.3% | 93.3% | 93.4% | 93.3% | 92.7% | 92.7% | 92.6% | 92.6% | 93.1% | 96.6% |
