# Supplementary material for: Continuing evolution of H6N2 influenza a virus in South African chickens and the implications for diagnosis and control
Source: BMC Vet Res. 2019 Dec 18;15:455. doi: 10.1186/s12917-019-2210-4 (PMC6921544; doi:10.1186/s12917-019-2210-4)
Supplement: Supplementary file 2 — Additional file 2: Table S1b. Percentage amino sequence identity in the HA proteins of all sub-lineage I viruses. [file 12917_2019_2210_MOESM2_ESM.docx]

**Table S1b. Percentage amino sequence identity in the HA proteins of all sub-lineage I viruses**

| Isolate | **W-04**  **/02** | **AL19**  **/02** | **NWY**  **/12** | **MAS**  **/13** | **338087**  **/15** | **339678**  **/15** | **341797**  **/15** | **344378**  **/15** | **344579**  **/15** | **398997**  **/16** | **401156**  **/16** | **402385**  **/16** | **404573**  **/16** | **N2826**  **/16** | **H44954**  **/16** |
| --- | --- | --- | --- | --- | --- | --- | --- | --- | --- | --- | --- | --- | --- | --- | --- |
| AL19/02 | 99.6% |  |  |  |  |  |  |  |  |  |  |  |  |  |  |
| NWY/12 | 92.6% | 92.6% |  |  |  |  |  |  |  |  |  |  |  |  |  |
| MAS/13 | 92.0% | 92.0% | 98.9% |  |  |  |  |  |  |  |  |  |  |  |  |
| 338087/15 | 92.0% | 92.0% | 98.0% | 97.3% |  |  |  |  |  |  |  |  |  |  |  |
| 339678/15 | 90.7% | 90.7% | 97.9% | 96.5% | 96.3% |  |  |  |  |  |  |  |  |  |  |
| 341797/15 | 90.7% | 90.7% | 96.9% | 96.3% | 96.1% | 99.8% |  |  |  |  |  |  |  |  |  |
| 344378/15 | 91.8% | 91.8% | 97.8% | 97.1% | 99.8% | 96.5% | 96.3% |  |  |  |  |  |  |  |  |
| 344579/15 | 91.6% | 91.6% | 97.5% | 96.7% | 99.5% | 96.1% | 95.9% | 99.6% |  |  |  |  |  |  |  |
| 398997/16 | 90.7% | 90.7% | 96.5% | 96.3% | 95.8% | 99.5% | 99.3% | 95.9% | 95.6% |  |  |  |  |  |  |
| 401156/16 | 90.9% | 90.9% | 97.1% | 96.5% | 96.3% | 99.3% | 99.1% | 96.5% | 96.1% | 98.7% |  |  |  |  |  |
| 402385/16 | 90.9% | 90.9% | 97.1% | 96.5% | 96.3% | 99.3% | 99.1% | 96.5% | 96.1% | 98.7% | 100% |  |  |  |  |
| 404573/16 | 90.7% | 90.7% | 96.5% | 96.3% | 95.8% | 99.5% | 99.3% | 95.9% | 95.6% | 100% | 98.7% | 98.7% |  |  |  |
| N2826/16 | 90.5% | 90.5% | 96.9% | 96.3% | 96.1% | 99.8% | 99.6% | 96.3% | 95.9% | 99.3% | 99.1% | 99.1% | 99.3% |  |  |
| H44954/16 | 90.9% | 90.9% | 96.9% | 96.3% | 95.9% | 95.4% | 95.2% | 96.1% | 95.9% | 94.8% | 95.4% | 95.4% | 94.8% | 95.6% |  |
| 432/19 | 90.5% | 90.5% | 95.9% | 95.4% | 95.0% | 94.4% | 94.2% | 95.2% | 95.0% | 93.8% | 94.4% | 94.4% | 93.9% | 94.2% | 96.7% |
