# Supplementary material for: Continuing evolution of H6N2 influenza a virus in South African chickens and the implications for diagnosis and control
Source: BMC Vet Res. 2019 Dec 18;15:455. doi: 10.1186/s12917-019-2210-4 (PMC6921544; doi:10.1186/s12917-019-2210-4)
Supplement: Supplementary file 3 — Additional file 3: Table S1c. Amino acid between-group distances. [file 12917_2019_2210_MOESM3_ESM.docx]

**Table S1c. Amino acid between-group distances**

|  | **2002** | **2012-2013** | **2015-2016** |
| --- | --- | --- | --- |
| 2012-2013 (n=3) | 96.1% |  |  |
| 2015-2016 (n=11) | 95.6% | 96.7% |  |
| 2019 (n=1) | 90.5% | 95.7% | 94.7% |
