# Supplementary material for: Continuing evolution of H6N2 influenza a virus in South African chickens and the implications for diagnosis and control
Source: BMC Vet Res. 2019 Dec 18;15:455. doi: 10.1186/s12917-019-2210-4 (PMC6921544; doi:10.1186/s12917-019-2210-4)
Supplement: Supplementary file 4 — Additional file 4: Figure S1. Alignment of the hemagglutinin protein sequences of South African H6N2 isolates from chickens. [file 12917_2019_2210_MOESM4_ESM.docx]

Figure S1. Alignment of the hemagglutinin protein sequences of South African H6N2 isolates from chickens. Corresponding H3 numbering is shown on the scale in red; amino acid identities are plotted to the first sequence with a dot; the red arrow indicates the HA_0_ cleavage site; strains from the present study are in bold. Predicted N-glycosylation sites are shaded in yellow; predicted O-glycosylation sites are shaded in green (isolates sequenced here only).

6 16 26 36 46 56 66 76 86 96 106 116

H3..|....| ....|....| ....|....| ....|....| ....|....| ....|....| ....|....| ....|....| ....|....| ....|....| ....|....| ....|....| ....|....|

10 20 30 40 50 60 70 80 90 100 110 120 130

....|....| ....|....| ....|....| ....|....| ....|....| ....|....| ....|....| ....|....| ....|....| ....|....| ....|....| ....|....| ....|....|

AL25/2002(II) **MIAIIVIAIL** **ASAGKSDKIC** **IGYHANNSTT** **QVDTILEKNV** **TVTHSIELLE** **TQKEERFCKI** **LNKAPLNLGG** **CTIEGWILGN** **PQCDLLLGDQ** **SWSYIVERPT** **AQNGICYPGV** **LNEVEELKAL** **IVSGERVERF**

Ostrich KK98 **-------...** **..A.......** **..........** **..........** **.....V....** **..........** **.........G** **..........** **..........** **..........** **.........A** **..........** **......I...**

BKR2/2012(II) **..........** **..........** **..........** **..........** **..........** **..........** **......D..E** **..........** **..........** **..........** **.R........** **..........** **.G...K....**

BKR4/2012(II) **..........** **..........** **..........** **..........** **..........** **..........** **......D..E** **..........** **..........** **..........** **.R........** **..........** **.G...K....**

AL19/2002(I) **..........** **..T.......** **..........** **..........** **..........** **N.........** **......D.RE** **..........** **..........** **..........** **.........P** **..........** **.G........**

W-04/2002(I) **..........** **..T.......** **..........** **..........** **..........** **N.........** **......D.RE** **..........** **..........** **..........** **.........P** **..........** **.G........**

UP1102/2002(I) **..........** **..........** **..........** **..........** **..........** **..........** **..........** **..........** **....S.....** **..........** **..........** **..........** **.G........**

MAS/2013(I) **.....A..L.** **V.T.......** **..........** **.........I** **..........** **....G...R.** **......D.RE** **......M...** **.H.....E..** **R........S** **.........P** **.......RS.** **.G....I...**

NWY/2012(I) **.....A..L.** **V.T.......** **..........** **.........I** **..........** **....G...R.** **......D.RE** **......M...** **.......E..** **R........S** **.........P** **.......RS.** **.G....I...**

**338087/2015(I)** **.....AV.L.** **V.T.......** **..........** **.........I** **..........** **........R.** **......D.RE** **......M...** **.......E..** **R........S** **.........P** **.......RTF** **.E........**

**339678/2015(I)** **.....A..L.** **V.T.......** **..........** **.........I** **..........** **........RV** **......D.RE** **......M...** **.R.....E..** **R........S** **.S.......P** **...I...RS.** **.G........**

**341797/2015(I)** **.....A..L.** **V.T.......** **..........** **.........I** **..........** **........RV** **......D.RE** **......M...** **.R.....E..** **R........S** **.S.......P** **...I...RS.** **.G........**

**344378/2015(I)** **.....AV.L.** **V.T.......** **..........** **.........I** **..........** **........R.** **......D.RE** **......M...** **.......E..** **R........S** **.........P** **.......RTF** **.E........**

**344579/2015(I)** **.....AV.L.** **V.T.......** **..........** **.........I** **..........** **........R.** **......D.RE** **......V...** **.......E..** **R........S** **.........P** **.......RTF** **.E........**

**398997/2016(I)** **.....A..L.** **..T.......** **..........** **.........I** **..........** **........RV** **......D.RE** **......M...** **.R.....E..** **R........S** **.S.......P** **...I...RS.** **.G........**

**401156/2016(I**) **.....A..L.** **V.T.......** **..........** **.........I** **..........** **........RV** **......D.RE** **......M...** **.R.....D..** **R........S** **.S.......P** **...I...RS.** **.G........**

**402385/2016(I) .....A..L.** **V.T.......** **..........** **.........I** **..........** **........RV** **......D.RE** **......M...** **.R.....D..** **R........S** **.S.......P** **...I...RS.** **.G........**

**404573/2016(I)** **.....A..L.** **..T.......** **..........** **.........I** **..........** **........RV** **......D.RE** **......M...** **.R.....E..** **R........S** **.S.......P** **...I...RS.** **.G........**

**N2826/2016(I)** **.....A..L.** **V.T.......** **..........** **.........I** **..........** **........RV** **......D.RE** **......M...** **.R..I..E..** **R........S** **.S.......P** **...I...RS.** **.G........**

**H44954/2016(I**) **.....A..L.** **V.T.......** **..........** **.........I** **..........** **........R.** **......D.RE** **......M...** **....I..E..** **R........S** **.........P** **.......RS.** **.G........**

**432/2019(I)**  **........L.** **V.T.......** **..........** **.........I** **..........** **........R.** **......D.RE** **......M...** **.......E..** **R........S** **.........P** **.......RS.** **.G........**

130 loop

190 helix

220 loop (223-228)

126 136 146 156 166 176 186 196 206 216 226 236 246

....|....| ....|....| ....|....| ....|....| ....|....| ....|....| ....|....| ....|....| ....|....| ....|....| ....|....| ....|....| ....|....|

140 150 160 170 180 190 200 210 220 230 240 250 260

....|....| ....|....| ....|....| ....|....| ....|....| ....|....| ....|....| ....|....| ....|....| ....|....| ....|....| ....|....| ....|....|

AL25/2002(II) **EMFPRSTWRG** **VDTNSGVTKA** **CPSSTGGSSF** **YRNLLWIIKN** **KSAAYPVIKG** **TYNNTGNQPI** **LYFWGVHHPP** **DTNEQNNLYG** **SGDRYVRMGT** **ESMNFAKSPE** **IAARPAVNGQ** **RGRIDYYWSV** **LNPGETLNVE**

Ostrich KK98 **..........** **...S..V...** **.P--NS....** **.........T** **..........** **..........** **..........** **...E..T...** **..........** **..........** **..........** **..........** **..........**

BKR2/2012(II) **.....N....** **..........** **..........** **.........S** **..........** **..........** **..........** **......T...** **..........** **.......G..** **..........** **..........** **.K........**

BKR4/2012(II) **.....N....** **..........** **..........** **.........S** **..........** **..........** **..........** **......T...** **..........** **.......G..** **..........** **..........** **.K........**

AL19/2002(I) **....K...T.** **...D..I...** **.S........** **..........** **..........** **..........** **..........** **...G......** **..........** **..........** **..........** **..........** **..........**

W-04/2002(I) **....K...T.** **...D..I...** **.-........** **..........** **..........** **..........** **..........** **...G......** **..........** **..........** **..........** **..........** **..........**

UP1102/2002(I) **..........** **..........** **..........** **.........S** **..........** **..........** **..........** **..H.......** **..........** **......Q...** **..........** **..........** **..........**

MAS/2013(I) **....K...T.** **...DN.I.R.** **.S........** **..........** **...S......** **A.........** **I.........** **.ADR......** **..........** **...H......** **.....S....** **..........** **..........**

NWY/2012(I) **....K...T.** **...DN.I.R.** **.S........** **..........** **...S......** **..........** **I.........** **.ADR......** **..........** **...H......** **.....S....** **..........** **..........**

**338087/2015(I) ....K...T.** **...DN.I.R.** **.S........** **..........** **...S......** **..........** **I.........** **.ADR......** **P......L..** **...H......** **.....S....** **..........** **..........**

**339678/2015(I) ....K...N.** **...EN.I.R.** **.S........** **..........** **...S......** **..........** **I.........** **.ADR......** **.....I....** **...H...G..** **.....S....** **..........** **........I.**

**341797/2015(I) ....K...N.** **...EN.I.R.** **.S........** **..........** **...P......** **..........** **I.........** **.ADR......** **.....I....** **...H...G..** **.....S....** **..........** **........I.**

**344378/2015(I) ....K...T.** **...DN.I.R.** **.S........** **..........** **...S......** **..........** **I.........** **.ADR......** **P....I.L..** **...H......** **.....S....** **..........** **..........**

**344579/2015(I) ....K...T.** **...DN.I.R.** **.S........** **..........** **...S......** **......Y...** **I.........** **.ADR......** **P....I.L..** **...H......** **.....S....** **..........** **..........**

**398997/2016(I) ....K...N.** **...EN.I.R.** **.S........** **..........** **...S......** **..........** **I.........** **.ADR......** **.....I....** **...H...G..** **.....S....** **..........** **........I.**

**401156/2016(I**) **....K...N.** **...EN.I.R.** **.L........** **..........** **...S......** **..........** **I.........** **.ADR......** **.....I....** **...H......** **.....S....** **..........** **........I.**

**402385/2016(I) ....K...N.** **...EN.I.R.** **.L........** **..........** **...S......** **..........** **I.........** **.ADR......** **.....I....** **...H......** **.....S....** **..........** **........I.**

**404573/2016(I) ....K...N.** **...EN.I.R.** **.S........** **..........** **...S......** **..........** **I.........** **.ADR......** **.....I....** **...H...G..** **.....S....** **..........** **........I.**

**N2826/2016(I)** **....K...N.** **...EN.I.R.** **.S........** **..........** **...S......** **..........** **I.........** **.ADR......** **.....I....** **...H...G..** **.....S....** **..........** **........I.**

**H44954/2016(I**) **....K...T.** **...DN.I.I.** **.S........** **..........** **...S......** **......K...** **I.........** **.ADR......** **.....I....** **...H......** **...G.S....** **.........I** **..........**

**432/2019(I)**  **....KN..T.** **...DN.I.R.** **.S........** **..........** **...S......** **......K...** **..........** **.AER......** **.....I....** **...H......** **...G.S....** **..........** **..........**

256 266 276 286 296 306 316 326 336 346 356 366 376

....|....| ....|....| ....|....| ....|....| ....|....| ....|....| ....|....| ....|....| ....|....| ....|....| ....|....| ....|....| ....|....|

**↓**

270 280 290 300 310 320 330 340 350 360 370 380 390

....|....| ....|....| ....|....| ....|....| ....|....| ....|....| ....|....| ....|....| ....|....| ....|....| ....|....| ....|....| ....|....|

AL25/2002(II) **SNGNLIAPWY** **AYKFVSTNNK** **GAVFKSNLPI** **EDCHATCQTA** **AGVLKINKRF** **QNVSPLWIGE** **CPKYVKSKSL** **RLATGLRNVP** **QIETRGLFGA** **IAGFIEGGWT** **GMIDGWYGYH** **HENSQGSGYA** **ADRESTQKAI**

Ostrich KK98 **........W.** **..K.V.....** **..........** **.....I....** **..........** **..........** **.......E..** **..........** **...P......** **..........** **..........** **..........** **..........**

BKR2/2012(II) **..........** **.......S..** **.........V** **.....I....** **....RV....** **..........** **..........** **.....P....** **..........** **..........** **.L........** **..........** **.........V**

BKR4/2012(II) **..........** **.......S..** **.........V** **.....I....** **....RV....** **..........** **..........** **.....P....** **..........** **..........** **.L........** **..........** **.........V**

AL19/2002(I) **........R.** **..R.F.....** **..........** **.N.D.L...T** **....RT..T.** **..........** **..........** **..........** **..........** **..........** **..........** **..........** **..........**

W-04/2002(I) **........R.** **..R.F.....** **..........** **.N.D.L...T** **....RT..T.** **..........** **..........** **..........** **..........** **..........** **..........** **..........** **..........**

UP1102/2002(I) **..........** **..........** **..I.......** **.....I....** **..........** **..........** **..........** **..........** **..........** **..........** **..........** **..........** **..........**

MAS/2013(I) **....F...R.** **..R.F.K.K.** **.VI.......** **.N.D.K...T** **S...RT..T.** **.....Q....** **..........** **..........** **.V....I...** **..........** **..........** **..........** **...D......**

NWY/2012(I) **....F...R.** **..R.F...K.** **.VI.......** **...D.Q...T** **S...RT..T.** **.....Q....** **..........** **..........** **.V....I...** **..........** **..........** **..........** **...D......**

**338087/2015(I) ....F...R.** **..R.F...K.** **.VI...T...** **.N.D.Q...T** **S...RT..T.** **.....Q.V..** **..........** **..........** **.V....I...** **..........** **..........** **..........** **...D......**

**339678/2015(I) ....F...R.** **..R.F...K.** **.VI.......** **.N.D.Q...T** **L...RT..T.** **.....Q.T..** **..........** **..........** **.V....I...** **..........** **..........** **..........** **...D......**

**341797/2015(I) ....F...R.** **..R.F...K.** **.VI.......** **.N.D.Q...T** **L...RT..T.** **.....Q.T..** **..........** **..........** **.V....I...** **..........** **..........** **..........** **...D......**

**344378/2015(I) ....F...R.** **..R.F...K.** **.VI...T...** **.N.D.Q...T** **S...RT..T.** **.....Q.V..** **..........** **..........** **.V....I...** **..........** **..........** **..........** **...D......**

**344579/2015(I) ....F...R.** **..R.F...K.** **.VI...T...** **.N.D.Q...T** **S...RT..T.** **.....Q.V..** **..........** **..........** **.V....I...** **..........** **..........** **..........** **...D......**

**398997/2016(I) ....F...R.** **..R.F...K.** **.VI.......** **.N.D.K...T** **L...RT..T.** **.....Q.T..** **..........** **..........** **.V....I...** **..........** **..........** **..........** **...D......**

**401156/2016(I**) **....F...R.** **..R.F...K.** **.VI.......** **.N.D.Q...T** **L...RT..T.** **.....Q.T..** **..........** **..........** **.V....I...** **..........** **..........** **..........** **...D......**

**402385/2016(I) ....F...R.** **..R.F...K.** **.VI.......** **.N.D.Q...T** **L...RT..T.** **.....Q.T..** **..........** **..........** **.V....I...** **..........** **..........** **..........** **...D......**

**404573/2016(I) ....F...R.** **..R.F...K.** **.VI.......** **.N.D.K...T** **L...RT..T.** **.....Q.T..** **..........** **..........** **.V....I...** **..........** **..........** **..........** **...D......**

**N2826/2016(I)** **....F...R.** **..R.F...K.** **.VI.......** **.N.D.Q...T** **L...RT..T.** **.....Q.T..** **..........** **..........** **.V....I...** **..........** **..........** **..........** **...D......**

**H44954/2016(I**) **....F...R.** **..R.F.S.K.** **.II.......** **.N.D.Q...T** **S...RT..T.** **.....Q....** **..........** **........I.** **.VG...I...** **..........** **..........** **..........** **...D......**

**432/2019(I)**  **....F...R.** **..R.FNS.K.** **.VI.......** **.N.E.Q...N** **S...RT..T.** **.....Q....** **..........** **........I.** **.V....I...** **..........** **..........** **..........** **...D......**

386 396 406 416 426 436 446 456 466 476 486 496 506

....|....| ....|....| ....|....| ....|....| ....|....| ....|....| ....|....| ....|....| ....|....| ....|....| ....|....| ....|....| ....|....|

400 410 420 430 440 450 460 470 480 490 500 510 520

....|....| ....|....| ....|....| ....|....| ....|....| ....|....| ....|....| ....|....| ....|....| ....|....| ....|....| ....|....| ....|....|

AL25/2002(II) **DGITNKVNAI** **VDKMNTQFEA** **VDHEFSNLER** **RIGNLNKRME** **DGFLDVWTYN** **AELLVLLENE** **RTLDLHDANV** **KNLYEKVKSQ** **LRDNANDLGN** **GCFEFWHKCD** **NDCIESVKNG** **TYDYPKYQDE** **SRLNRQEIES**

Ostrich KK98 **........S.** **..........** **..........** **..........** **..........** **..........** **..........** **..--------** **----------** **----------** **----------** **----------** **----------**

BKR2/2012(II) **..........** **..........** **..........** **..........** **..........** **..........** **..........** **...F......** **..........** **..........** **...M......** **.....NH.E.** **.K........**

BKR4/2012(II) **..........** **..........** **..........** **..........** **..........** **..........** **..........** **...F......** **..........** **..........** **...M......** **.....NH.E.** **.K........**

AL19/2002(I) **..........** **I.........** **..........** **..D.......** **..........** **..........** **..........** **..........** **..........** **..........** **...M......** **......H...** **.K....K...**

W-04/2002(I) **..........** **I.........** **..........** **..D.......** **..........** **..........** **..........** **..........** **..........** **..........** **...M......** **..........** **.K........**

UP1102/2002(I) **..........** **..........** **..........** **..........** **..........** **..........** **..........** **.S........** **..........** **..........** **..........** **..........** **.K........**

MAS/2013(I) **........T.** **I.........** **.G........** **..D.......** **..L.......** **..........** **..........** **..........** **..........** **..........** **...M......** **..........** **.K....K...**

NWY/2012(I) **........T.** **I.........** **.G........** **..D.......** **..L.......** **..........** **..........** **..........** **..........** **..........** **...M......** **..........** **.K....K...**

**338087/2015(I) ........T.** **I.........** **.G........** **..D.......** **..L.......** **..........** **..........** **..........** **..........** **..........** **...M......** **..........** **.K....K...**

**339678/2015(I) ........T.** **I.........** **.G........** **..D.......** **..L.......** **..........** **..........** **.....R....** **..........** **..........** **...M......** **..........** **.K....K...**

**341797/2015(I) ........T.** **I.........** **.G........** **..D.......** **..L.......** **..........** **..........** **.....R....** **..........** **..........** **...M......** **..........** **.K....K...**

**344378/2015(I) ........T.** **I.........** **.G........** **..D.......** **..L.......** **..........** **..........** **..........** **..........** **..........** **...M......** **..........** **.K....K...**

**344579/2015(I) ........T.** **I.........** **.G........** **..D.......** **..L.......** **..........** **..........** **..........** **..........** **..........** **...M......** **..........** **.K....K...**

**398997/2016(I) ........T.** **I.........** **.G........** **..D.......** **..LM......** **..........** **..........** **.....R....** **..........** **..........** **...M......** **..........** **.K....K...**

**401156/2016(I**) **........T.** **I.........** **.G........** **..D.......** **..L.......** **..........** **..........** **..........** **..........** **..........** **...M......** **..........** **.K....K...**

**402385/2016(I) ........T.** **I.........** **.G........** **..D.......** **..L.......** **..........** **..........** **..........** **..........** **..........** **...M......** **..........** **.K....K...**

**404573/2016(I) ........T.** **I.........** **.G........** **..D.......** **..LM......** **..........** **..........** **.....R....** **..........** **..........** **...M......** **..........** **.K....K...**

**N2826/2016(I)** **........T.** **I.........** **.G........** **..D.......** **..L.......** **..........** **..........** **.....R....** **..........** **..........** **...M......** **..........** **.K....K...**

**H44954/2016(I**) **........S.** **I.........** **.G........** **..D.M.....** **..L.......** **..........** **..........** **..........** **..........** **..........** **...M......** **..........** **.K....K...**

**432/2019(I)**  **........S.** **IG........** **IG........** **..D.M.....** **..L.......** **....I.....** **..........** **..........** **..........** **..........** **...M......** **........N.** **.K....K...**

516 526 536 546

....|....| ....|....| ....|....| ....|....| ....|...

530 540 550 560

....|....| ....|....| ....|....| ....|....| ....|...

AL25/2002(II) **VKLENLGVYQ** **ILAIYSTVSS** **SLVLVGLIIA** **MGLWMCSNGS** **MQCRICI***

Ostrich KK98 **----------** **----------** **----------** **----------** **--------**

BKR2/2012(II) **..........** **..........** **..........** **I.........** **.......***

BKR4/2012(II) **..........** **..........** **..........** **I.........** **.......***

AL19/2002(I) **...D......** **..........** **..........** **..........** **.......***

W-04/2002(I) **...D......** **..........** **..........** **..........** **.......***

UP1102/2002(I) **....D.....** **..........** **..........** **..........** **..S....***

MAS/2013(I) **...D......** **..........** **.......V..** **..........** **.......***

NWY/2012(I) **...D......** **..........** **..........** **..........** **.......***

**338087/2015(I) ...D......** **..........** **..........** **..........** **.......***

**339678/2015(I) ...D......** **..........** **..........** **..........** **....V..***

**341797/2015(I) ...D......** **..........** **..........** **..........** **....V..***

**344378/2015(I) ...D......** **..........** **..........** **..........** **.......***

**344579/2015(I) ...D......** **..........** **..........** **..........** **.......***

**398997/2016(I) ...D......** **..........** **..........** **..........** **....V..***

**401156/2016(I**) **...D......** **..........** **..........** **..........** **....V..***

**402385/2016(I) ...D......** **..........** **..........** **..........** **....V..***

**404573/2016(I) ...D......** **..........** **..........** **..........** **....V..***

**N2826/2016(I)** **...D......** **..........** **..........** **..........** **....V..***

**H44954/2016(I**) **.N.D......** **..........** **........M.** **..........** **.......***

**432/2019(I)**  **...D......** **..........** **........L.** **..........** **.......***
