# Supplementary material for: Continuing evolution of H6N2 influenza a virus in South African chickens and the implications for diagnosis and control
Source: BMC Vet Res. 2019 Dec 18;15:455. doi: 10.1186/s12917-019-2210-4 (PMC6921544; doi:10.1186/s12917-019-2210-4)
Supplement: Supplementary file 5 — Additional file 5: Table S2. Predicted glycosylation patterns in the surface glycoproteins of H6N2 influenza viruses isolated since 2015. [file 12917_2019_2210_MOESM5_ESM.docx]

**Table S2. Predicted glycosylation patterns in the surface glycoproteins of H6N2 influenza viruses isolated since 2015**

| Strain | **Hemagglutinin protein** | | **Neuraminidase protein*** | |
| --- | --- | --- | --- | --- |
|  | **N-glycosylated residues** | **O-glycosylated residues** | **N-glycosylated residues** | **O-glycosylated residues** |
| 338087/2015 | 26, 27, 39, 170, 183, 307, 312, 499, 558 | 152, 314 | 64, 124, 178, 212, 287, 291, 380 | 123, 309, 311 |
| 341797/2015 | 26, 27, 39, 170, 183, 307, 312, 499, 558 | 152, 314 | 64, 124, 178, 212, 291, 380 | 123, 311 |
| 339678/2015 | 26, 27, 39, 170, 183, 307, 312, 499, 558 | 152, 314 | 64, 124, 178, 212, 291, 380 | 123, 311 |
| 344378/2015 | 26, 27, 39, 170, 183, 307, 312, 499, 558 | 152, 314 | 64, 124, 178, 212, 287, 291, 380 | 123, 309, 311 |
| 344579/2015 | 26, 27, 39, 170, 183, 307, 312, 499, 558 | 152, 314 | 64, 124, 178, 212, 287, 291, 380 | 123, 309, 311 |
| 398997/2016 | 26, 27, 39, 170, 183, 307, 312, 499, 558 | 152, 314 | 64, 124, 178, 212, 291, 380 | 123, 311 |
| 401156/2016 | 26, 27, 39, 170, 183, 307, 312, 499, 558 | 152, 314 | 64, 124, 178, 212, 291, 380 | 123, 311 |
| 402385/2016 | 26, 27, 39, 170, 183, 307, 312, 499, 558 | 152, 314 | 64, 124, 178, 212, 291, 380 | 123, 311 |
| 404573/2016 | 26, 27, 39, 170, 183, 307, 312, 499, 558 | 152, 314 | 64, 124, 178, 212, 287, 291, 380 | 123, 309, 311 |
| N2826/2016 | 27, 39, 170, 183, 307, 499, 558 | 152, 314 | 64, 124, 212 | 123, 311 |
| H44954/2016 | 26, 27, 39, 170, 183, 307, 312, 499, 558 | 152 | 54, 64, 124, 178, 212, 287, 291, 380 | 123, 309, 310, 311, 313 |
| 432/2019 | 26, 27, 39, 170, 183, 307, 312, 499, 509, 558 | 152, 314 | **63, 123, 177, 211, 286, 290, 379 | **122, 310 |

*numbering according to full-length NA

**numbering differs due to a codon deletion at position 46
