# Supplementary material for: Continuing evolution of H6N2 influenza a virus in South African chickens and the implications for diagnosis and control
Source: BMC Vet Res. 2019 Dec 18;15:455. doi: 10.1186/s12917-019-2210-4 (PMC6921544; doi:10.1186/s12917-019-2210-4)
Supplement: Supplementary file 6 — Additional file 6: Figure S2. Alignment of the neuraminidase protein sequences of South African H6N2 isolates from chickens. [file 12917_2019_2210_MOESM6_ESM.docx]

Figure S2. Alignment of the neuraminidase protein sequences of South African H6N2 isolates from chickens. Amino acid identities are plotted to the first sequence with a dot; (-) indicates a missing amino acid; *only partial sequences are available, not a true reflection of the N-stalk deletion in these isolates; strains from the present study are in bold. Predicted N-glycosylation sites are shaded in yellow; predicted O-glycosylation sites are shaded in green (isolates sequenced here only). HBS: HB site.

10 20 30 40 50 60 70 80 90 100 110 120 130

....|....| ....|....| ....|....| ....|....| ....|....| ....|....| ....|....| ....|....| ....|....| ....|....| ....|....| ....|....| ....|....|

9508103/1995 **MNPNQKIITI** **GSVSLTIATI** **CFLMQIAILA** **TTVTLHFKQN** **GCSIPSNNQV** **VPCEPIIVER** **NITEIVYLNN** **TTIEKELCPK** **VLEYRDWSKP** **QCQITGFAPF** **SKDNSIRLSA** **GGDIWITREP** **YVSCSPDKCY**

AL25/2002(II) **..........** **.....I....** **..........** **..........** **....S.....** **.......I..** **..........** **......F...** **A......L..** **..........** **..........** **.....V....** **......E...**

BKR2/2012(II) **..........** **..........** **..........** **.........G** **.....V....** **.....V.I.K** **...K......** **.I....F...** **A....N.L..** **..........** **..........** **.....V....** **......E...**

BKR4/2012(II) **..........** **..........** **..........** **.........G** **.....V....** **.....V.I.K** **...K......** **.I....F...** **A....N.L..** **..........** **..........** **.....V....** **......E...**

BKP/2012(II) **..........** **..........** **..........** **.........G** **.....V....** **.....V.I.K** **...K......** **.I....F...** **A....N.L..** **..........** **..........** **.....V....** **......E...**

UP1102/2002(II) **..........** **..........** **..........** **.......E..** **......T...** **.......I..** **..........** **......S...** **A....E.L..** **..........** **..........** **.....V....** **......E...**

AL19/2002(I)* **..........** **..........** **..........** **M....Q----** **----------** **----------** **----------** **----------** **-......L..** **..........** **..........** **.....A....** **......E...**

W-04/2002(I)* **..........** **..........** **..........** **..I..Q----** **----------** **----------** **----------** **----------** **-......L..** **..........** **..........** **.....V....** **......E...**

NWY/2012(I)* **..........** **..........** **..........** **.....Q----** **----------** **----------** **----------** **----------** **-....N....** **..........** **..........** **.....V....** **......E...**

MAS/2013(I) **..........** **..........** **..........** **..........** **.Y.......A** **......----** **----------** **----------** **-....N....** **..........** **..........** **.....V....** **......E...**

**338087/2015(I)**  **..........** **..........** **.......T.V** **..I.......** **.Y....T..A** **......L---** **----------** **---------T** **T....N....** **..........** **..........** **.....V....** **......E...**

**339678/2015(I)**  **..........** **.......T..** **..........** **..........** **.Y.......A** **......L---** **----------** **---------.** **T....N....** **..........** **..........** **.....V....** **......E..F**

**341797/2015(I)**  **..........** **.......T..** **..........** **...M.....S** **.Y.......A** **......L---** **----------** **---------.** **T....N....** **..........** **..........** **.....V....** **......E..F**

**344378/2015(I)**  **..........** **..........** **.......T.V** **..I.......** **.Y....T..A** **......L---** **----------** **---------T** **T....N....** **..........** **..........** **.....V....** **......E...**

**344579/2015(I)**  **..........** **..........** **.......T.V** **..I.......** **.Y....T..A** **......L---** **----------** **---------T** **T....N....** **..........** **..........** **.....V....** **......E...**

**398997/2016(I)**  **..........** **.......T..** **..........** **..........** **.Y...P...A** **......P---** **----------** **---------.** **T....N....** **..........** **..........** **.....V....** **......E..F**

**401156/2016(I)**  **..........** **.......T..** **..........** **..........** **.Y.......A** **......L---** **----------** **---------.** **T....N....** **..........** **..........** **.....V....** **......E..F**

**402385/2016(I)**  **..........** **.......T..** **..........** **..........** **.Y.......A** **......L---** **----------** **---------.** **T....N....** **..........** **..........** **.....V....** **......E..F**

**404573/2016(I)**  **..........** **..........** **.......T.V** **..I...L...** **EY....T..A** **.....TF---** **----------** **---------T** **T....N....** **..........** **..........** **.....V....** **......E...**

**H44954/2016(I)**  **..........** **..........** **..........** **...M......** **.Y.......A** **...N.TL---** **----------** **---------.** **T....N....** **..........** **..........** **.....V....** **......E...**

**N2826/2016(I)**  **..........** **.......T..** **..........** **..........** **.Y...P...A** **......P---** **----------** **---------.** **T....N....** **.......V..** **..........** **.....V....** **......E..F**

**432/2019(I)**  **..........** **.......T..** **..........** **..........** **.Y.V.-.T.A** **......P---** **----------** **---------.** **TS...N....** **..........** **..........** **.....V....** **......E..F**

140 150 160 170 180 190 200 210 220 230 240 250 260

....|....| ....|....| ....|....| ....|....| ....|....| ....|....| ....|....| ....|....| ....|....| ....|....| ....|....| ....|....| ....|....|

9508103/1995 **QFALGQGTTL** **DNKHSNGTIH** **DRIPHRTLLM** **NELGVPFHLG** **TKQVCIAWSS** **SSCHDGKAWL** **HVCVTGDDRN** **ATASFIYDGV** **LVDSIGSWSQ** **NILRTQESEC** **VCINGTCAVV** **MTDGSASGRA** **DTRILFIKEG**

AL25/2002(II) **..........** **..........** **..........** **S.........** **..........** **..........** **..........** **.......N.L** **..........** **..........** **.......T..** **..........** **..........**

BKR2/2012(II) **..........** **H.........** **..V.......** **..........** **..........** **..........** **........G.** **.......N..** **.........R** **..........** **.......T..** **..........** **..........**

BKR4/2012(II) **..........** **H.........** **..V.......** **..........** **..........** **..........** **........G.** **.......N..** **.........R** **..........** **.......T..** **..........** **..........**

BKP/2012(II) **..........** **H.........** **..V.......** **..........** **..........** **..........** **........G.** **.......N..** **.........R** **..........** **.......T..** **..........** **..........**

UP1102/2002(II) **..........** **..........** **..........** **S.........** **..........** **..........** **..........** **.......N.L** **..........** **..........** **.......T..** **........M.** **..........**

AL19/2002(I) **..........** **..Q.......** **..T.......** **..........** **..........** **..........** **..........** **.......N..** **..........** **..........** **.......T..** **..........** **..........**

W-04/2002(I) **H.........** **..Q.......** **..T.......** **..........** **..........** **..........** **..........** **.......N..** **..........** **..........** **.......T..** **..........** **..........**

NWY/2012(I) **.......A..** **E.Q.......** **..T.......** **....I.....** **..........** **..........** **..........** **.......N..** **....V.....** **..........** **.......T..** **..........** **.......R..**

MAS/2013(I) **.......A..** **E.Q.......** **..T.......** **....I.....** **..........** **..........** **..........** **.......N..** **....V.....** **..........** **.......T..** **..........** **.......R..**

**338087/2015(I)**  **.......A..** **E.Q.......** **..T.......** **....I.....** **..........** **..........** **..........** **.......N..** **....V.....** **..........** **.......T..** **..........** **.......R..**

**339678/2015(I)**  **.......A..** **K.Q.......** **..T.......** **....I.....** **..........** **..........** **..........** **.......N..** **....V.....** **..........** **.......T..** **..........** **.......R..**

**341797/2015(I)**  **.......A..** **K.Q.......** **..T.......** **....I.....** **..........** **..........** **..........** **.......N..** **....V.....** **..........** **.......T..** **.......E..** **.......R..**

**344378/2015(I)**  **.......A..** **E.Q.......** **..T.......** **....I.....** **..........** **..........** **..........** **.......N..** **....V.....** **..........** **.......T..** **..........** **.......R..**

**344579/2015(I)**  **.......A..** **E.Q.......** **..T.......** **....I.....** **..........** **..........** **..........** **.......N..** **....V.....** **..........** **.......T..** **..........** **.......R..**

**398997/2016(I)**  **.......A..** **K.Q.......** **..T.......** **....I.....** **..........** **..........** **..........** **.......N..** **....V.....** **..........** **.......T..** **..........** **.......R..**

**401156/2016(I)**  **.......A..** **K.Q.......** **..T.......** **....I.....** **..........** **..........** **..........** **.......N..** **....V.....** **..........** **.......T..** **..........** **.......R..**

**402385/2016(I)**  **.......A..** **K.Q.......** **..T.......** **....I.....** **..........** **..........** **..........** **.......N..** **....V.....** **..........** **.......T..** **..........** **.......R..**

**404573/2016(I)**  **.......A..** **G.Q.......** **..T.......** **....I.....** **..........** **..........** **..........** **.......N..** **....V.....** **..........** **.......T..** **..........** **.......R..**

**H44954/2016(I)**  **.......A..** **G.Q.......** **..T.......** **....I.....** **..........** **..........** **..........** **.......N..** **....V.....** **..........** **.......T..** **..........** **..........**

**N2826/2016(I)**  **.......A..** **K.Q.......** **..T.......** **....I.....** **..........** **..........** **..........** **.......N..** **....V.....** **..........** **.......T..** **..........** **.......R..**

**432/2019(I)**  **.......A..** **K.Q.......** **..T.......** **....I.....** **..........** **..........** **..........** **.......N..** **....V.....** **..........** **.......T..** **........K.** **.......R..**

HBS

270 280 290 300 310 320 330 340 350 360 370 380 390

....|....| ....|....| ....|....| ....|....| ....|....| ....|....| ....|....| ....|....| ....|....| ....|....| ....|....| ....|....| ....|....|

9508103/1995 **KIVHVSPLSG** **SAQHIEECSC** **YPRYPDVKCV** **CRDNWKGSNR** **PIIDINVADY** **SIDSSYVCSG** **LVGDTPRNDD** **SSSNSNCKDP** **NNERGNPGVK** **GWAFDYGNDV** **WMGRTISKDS** **RSGYETFRVI** **GGWTTANSKS**

AL25/2002(II) **....I.....** **..........** **.......R..** **..........** **......M...** **..........** **..........** **.......R..** **..........** **.......S..** **..........** **..........** **..........**

BKR2/2012(II) **....I.....** **..........** **..H..N.R..** **..........** **..V...M...** **..........** **..........** **.T........** **..........** **..........** **..........** **..........** **..........**

BKR4/2012(II) **....I.....** **..........** **..H..N.R..** **..........** **..V...M...** **..........** **..........** **.T........** **..........** **..........** **..........** **..........** **..........**

BKP/2012(II) **....I.....** **..........** **..H..N.R..** **..........** **..V...M...** **..........** **..........** **.T........** **..........** **..........** **..........** **..........** **..........**

UP1102/2002(II) **....I.....** **..........** **.......R.I** **..........** **......M...** **..........** **..........** **.F.....R..** **..........** **.......S..** **..........** **..........** **..........**

AL19/2002(I) **.V..I.....** **G...T.....** **.......R..** **..........** **......M...** **..........** **..........** **.......R..** **..........** **..........** **..........** **..........** **..........**

W-04/2002(I) **....I.....** **G...T.....** **.......R..** **..........** **......M...** **..........** **..........** **.......R..** **..........** **..........** **..........** **..........** **..........**

NWY/2012(I) **..I.T.....** **G.........** **.......R..** **..........** **......M.N.** **..N.......** **..........** **.......R..** **...K......** **......ED..** **..........** **.......K..** **...I......**

MAS/2013(I) **..I.T.....** **G.........** **.......R..** **..........** **......M.N.** **..N.......** **..........** **.......R..** **...K......** **......ED..** **..........** **.......K..** **...I......**

**338087/2015(I)**  **..I.T.....** **G.........** **...H...R..** **..........** **......M.N.** **..N.......** **..........** **.......R..** **...K......** **......ED..** **..........** **.......K..** **..........**

**339678/2015(I)**  **....T.....** **..........** **.......R..** **..........** **......M.N.** **G.N.......** **..........** **D......R..** **..........** **.......D..** **..........** **.......K..** **...I......**

**341797/2015(I)**  **....T.....** **..........** **.......R..** **..........** **......M.N.** **G.N.......** **..........** **D......R..** **..........** **.......D..** **..........** **.......K..** **...I......**

**344378/2015(I)**  **..I.T.....** **G.........** **...H...R..** **..........** **......M.N.** **..N.......** **..........** **.......R..** **...K......** **......ED..** **..........** **.......K..** **..........**

**344579/2015(I)**  **..I.T.....** **G.........** **...H...R..** **..........** **......M.N.** **..N.......** **..........** **.......R..** **...K......** **......ED..** **..........** **.......K..** **..........**

**398997/2016(I)**  **....T.....** **..........** **.......R..** **..........** **......M.N.** **G.N.......** **..........** **D......R..** **..........** **.......D..** **..........** **.......K..** **...I......**

**401156/2016(I)**  **....T.....** **..........** **.......R..** **..........** **......M.N.** **G.N.......** **..........** **G......R..** **..........** **.......D..** **..........** **.......K..** **...I......**

**402385/2016(I)**  **....T.....** **..........** **.......R..** **..........** **......M.N.** **G.N.......** **..........** **D......R..** **..........** **.......D..** **..........** **.......K..** **...I......**

**404573/2016(I)**  **..I.T.....** **G.........** **...H...R..** **..........** **......M.N.** **..N.......** **..........** **.......R..** **...K......** **......ED..** **..........** **.......K..** **..........**

**H44954/2016(I)**  **..I.T.....** **G.L.V.....** **.......R..** **..........** **......M.N.** **..N.......** **.......H..** **.......R..** **......S...** **......ED..** **..........** **.......K..** **...IK.....**

**N2826/2016(I)**  **....T.....** **..........** **.......R..** **..........** **......M.N.** **G.N.......** **..........** **D......R..** **..........** **.......D..** **..........** **.......K..** **...I......**

**432/2019(I)**  **....I.....** **..........** **.......R..** **..........** **.V....MTN.** **D.N.......** **..........** **D......R..** **..........** **.....C.D..** **..........** **.......K..** **...IQ.....**

HBS

HBS

400 410 420 430 440 450 460

....|....| ....|....| ....|....| ....|....| ....|....| ....|....| ....|....| ....

9508103/1995 **QVNRQVIVDN** **NNWSGYSGIF** **SVEGKSCINR** **CFYVELIRGR** **PQETRVWWTS** **NSIVVFCGTS** **GTYGTGSWPD** **GANI**

AL25/2002(II) **..........** **..........** **..........** **..........** **..........** **..........** **..........** **....**

BKR2/2012(II) **..........** **..........** **..........** **..........** **..........** **..........** **..........** **....**

BKR4/2012(II) **..........** **..........** **..........** **..........** **..........** **..........** **..........** **....**

BKP/2012(II) **..........** **..........** **..........** **..........** **..........** **..........** **..........** **....**

UP1102/2002(II) **..........** **..........** **..........** **..........** **..........** **..........** **..........** **....**

AL19/2002(I) **..........** **..........** **...S......** **..........** **..........** **..........** **..........** **....**

W-04/2002(I) **..........** **..........** **...S......** **..........** **..........** **..........** **..........** **....**

NWY/2012(I) **..........** **........V.** **...R......** **..........** **.R........** **..........** **..........** **....**

MAS/2013(I) **..........** **........V.** **...R......** **..........** **.R........** **..........** **..........** **....**

**338087/2015(I)**  **..........** **........V.** **...R......** **..........** **.R........** **..........** **..........** **....**

**339678/2015(I)**  **..........** **........V.** **...R......** **..........** **.R.A......** **..........** **..........** **....**

**341797/2015(I)**  **..........** **........V.** **...R......** **..........** **.R.A......** **..........** **..........** **....**

**344378/2015(I)**  **..........** **........V.** **...R......** **..........** **.R........** **..........** **..........** **....**

**344579/2015(I)**  **..........** **........V.** **...R......** **..........** **.R........** **..........** **..........** **....**

**398997/2016(I)**  **..........** **........V.** **...R.G....** **..........** **.R.A......** **..........** **..........** **....**

**401156/2016(I)**  **..........** **........V.** **...RRG....** **..........** **.R.A......** **..........** **..........** **....**

**402385/2016(I)**  **..........** **........V.** **...RR.....** **..........** **.R.A......** **..........** **..........** **....**

**404573/2016(I)**  **..........** **........V.** **...R......** **..........** **.R........** **..........** **..........** **....**

**H44954/2016(I)**  **..........** **D.......V.** **...R......** **...I......** **..........** **..........** **..........** **....**

**N2826/2016(I)**  **..........** **........V.** **...R.G....** **..........** **.R.A......** **..........** **..........** **....**

**432/2019(I)**  **..........** **..........** **...R.N...M** **..........** **.R.A......** **..........** **..........** **....**
