# Supplementary material for: Continuing evolution of H6N2 influenza a virus in South African chickens and the implications for diagnosis and control
Source: BMC Vet Res. 2019 Dec 18;15:455. doi: 10.1186/s12917-019-2210-4 (PMC6921544; doi:10.1186/s12917-019-2210-4)
Supplement: Supplementary file 7 — Additional file 7: Figure S3. Alignment of the polymerase B2 (PB2) protein sequences of South African H6N2 isolates from chickens. [file 12917_2019_2210_MOESM7_ESM.docx]

**Figure S3.** Alignment of the polymerase B2 (PB2) protein sequences of South African H6N2 isolates from chickens. Amino acid identities are plotted to the first sequence with a dot; strains from the present study are in bold.

10 20 30 40 50 60 70 80 90 100 110 120 130

....|....| ....|....| ....|....| ....|....| ....|....| ....|....| ....|....| ....|....| ....|....| ....|....| ....|....| ....|....| ....|....|

KK98/1998 **MERIKELRDL** **ISQSRTREIL** **TKTTVDHMAI** **IKKYTSGRQE** **KNPALRMKWM** **MAMKYPITAD** **KRIMEMIPER** **NEQGQTLWSK** **TNDAGSDRVM** **VSPLAVTWWN** **RNGPTTSTVH** **YPKVYKTYFE** **KVERLKHGTF**

AL25/2002(II) **..........** **M.........** **..........** **..........** **..........** **..........** **..V.......** **..........** **..........** **..........** **..........** **..........** **..........**

BKR2/2012(II) **..........** **M.........** **..........** **..........** **..........** **..........** **..........** **..........** **..........** **..........** **........I.** **..........** **..........**

BKR4/2012(II) **..........** **M.........** **..........** **..........** **..........** **..........** **..........** **..........** **..........** **..........** **........I.** **..........** **..........**

BKP/2012(II) **..........** **M.........** **..........** **..........** **..........** **..........** **..........** **..........** **..........** **..........** **........I.** **..........** **..........**

AL19/2002(I) **..........** **M.........** **..........** **..........** **..........** **..........** **..V.......** **..........** **..........** **..........** **..........** **..........** **..........**

W-04/2002(I) **..........** **M.........** **..........** **..........** **..........** **..........** **..V.......** **..........** **..........** **..........** **..........** **......P...** **..........**

NWY/2012(I) **..........** **M.........** **..........** **..........** **..........** **..........** **..........** **..........** **..........** **..........** **........I.** **..........** **..........**

MAS/2013(I) **..........** **M.........** **..........** **..........** **..........** **..........** **..........** **..........** **..........** **..........** **........I.** **..........** **..........**

**338087/2015(I)**  **..........** **M.........** **.Q........** **..........** **..........** **..........** **..........** **..........** **..........** **..........** **........I.** **..........** **......N...**

**341797/2015(I)**  **..........** **M.........** **..........** **..........** **..........** **..........** **..........** **..........** **.T........** **..........** **.......AI.** **..........** **..........**

**344378/2015(I)**  **..........** **M.........** **.Q........** **..........** **..........** **..........** **..........** **..........** **..........** **..........** **........I.** **..........** **......N...**

**344579/2015(I)**  **..........** **M.........** **.Q........** **..........** **..........** **..........** **..........** **..........** **..........** **..........** **........I.** **..........** **......N...**

**398997/2016(I)**  **..........** **M.........** **..........** **..........** **..........** **..........** **..........** **..........** **.T........** **..........** **.......AI.** **..........** **..........**

**401156/2016(I)**  **..........** **M.........** **..........** **..........** **..........** **..........** **..........** **..........** **.T........** **..........** **.......VI.** **..........** **..........**

**402385/2016(I)**  **..........** **M.........** **..........** **..........** **..........** **..........** **..........** **..........** **.T........** **..........** **.......VI.** **..........** **..........**

**404573/2016(I)**  **..........** **M.........** **..........** **..........** **..........** **..........** **..........** **..........** **.T........** **..........** **.......AI.** **..........** **..........**

**N2826/2016(I)**  **..........** **M.........** **..........** **..........** **..........** **..........** **..........** **..........** **.T........** **..........** **.......AI.** **..........** **..........**

**H44954/2016(I)**  **..........** **M.........** **..........** **..........** **..........** **..........** **..........** **..........** **..........** **..........** **........I.** **..........** **........I.**

**432/2019(I)**  **..........** **M.........** **..........** **..........** **..........** **..........** **..........** **..........** **.T......L.** **..........** **.......AI.** **..........** **..........**

140 150 160 170 180 190 200 210 220 230 240 250 260

....|....| ....|....| ....|....| ....|....| ....|....| ....|....| ....|....| ....|....| ....|....| ....|....| ....|....| ....|....| ....|....|

KK98/1998 **GPVHFRNQVK** **IRRRVDINPG** **HADLSAKEAQ** **DVIMEVVFPN** **EVGARILTSE** **SQLTITKEKK** **EELQDCKIAP** **LMVAYMLERE** **LVRKTRFLPV** **AGGTSSVYIE** **VLHLTQGTCW** **EQMYTPGGDV** **RNDDVDQSLI**

AL25/2002(II) **..........** **..........** **..........** **..........** **..........** **.......V..** **..........** **..........** **..........** **..........** **..........** **........E.** **..........**

BKR2/2012(II) **..........** **..........** **..........** **..........** **..........** **...A...V..** **..........** **..........** **..........** **..........** **..........** **........E.** **.........V**

BKR4/2012(II) **..........** **..........** **..........** **..........** **..........** **...A...V..** **..........** **..........** **..........** **..........** **..........** **........E.** **.........V**

BKP/2012(II) **..........** **..........** **..........** **..........** **..........** **...A...V..** **..........** **..........** **..........** **..........** **..........** **........E.** **.........V**

AL19/2002(I) **..........** **..........** **..........** **..........** **..........** **.......V..** **..........** **..........** **..........** **..........** **..........** **........E.** **..........**

W-04/2002(I) **..........** **..........** **..........** **..........** **..........** **..........** **..........** **..........** **..........** **..........** **..........** **........E.** **..........**

NWY/2012(I) **..........** **..........** **..........** **..........** **..........** **...A...V..** **..........** **..........** **..........** **..........** **..........** **........E.** **.........V**

MAS/2013(I) **..........** **..........** **..........** **..........** **.......A..** **...A...V..** **..........** **..........** **..........** **..........** **..........** **........E.** **.........V**

**338087/2015(I)**  **..........** **..........** **..........** **..........** **..........** **...A..RI..** **..........** **..........** **..........** **..........** **..........** **........K.** **.........V**

**341797/2015(I)**  **..........** **..........** **..........** **..........** **..........** **...A...I..** **....N.....** **..........** **..........** **..........** **..........** **........E.** **..........**

**344378/2015(I)**  **..........** **..........** **..........** **..........** **..........** **...A..RI..** **..........** **..........** **..........** **..........** **..........** **........K.** **.........V**

**344579/2015(I)**  **..........** **..........** **.......A..** **..........** **..........** **...A..RI..** **..........** **..........** **..........** **..........** **..........** **........K.** **.........V**

**398997/2016(I)**  **..........** **..........** **..........** **..........** **..........** **...A...I..** **....N.....** **..........** **..........** **..........** **..........** **........E.** **..........**

**401156/2016(I)**  **..........** **..........** **..........** **..........** **..........** **...A...I..** **....N.....** **..........** **..........** **..........** **..........** **........E.** **..........**

**402385/2016(I)**  **..........** **..........** **..........** **..........** **..........** **...A...I..** **....N.....** **..........** **..........** **..........** **..........** **........E.** **..........**

**404573/2016(I)**  **..........** **..........** **..........** **..........** **..........** **...A...I..** **....N.....** **..........** **..........** **..........** **..........** **........E.** **....L.....**

**N2826/2016(I)**  **..........** **..........** **..........** **..........** **..........** **...A...I..** **....N.....** **..........** **..........** **..........** **..........** **........E.** **..........**

**H44954/2016(I)**  **..........** **..........** **..........** **..........** **..........** **...A...T..** **K.........** **..........** **..........** **..........** **..........** **........E.** **..........**

**432/2019(I)**  **..........** **..........** **..........** **..........** **..........** **...A...I..** **....N.....** **..........** **..........** **..........** **..........** **........E.** **..........**

270 280 290 300 310 320 330 340 350 360 370 380 390

....|....| ....|....| ....|....| ....|....| ....|....| ....|....| ....|....| ....|....| ....|....| ....|....| ....|....| ....|....| ....|....|

KK98/1998 **IAARNIVRRA** **TVSADPLASL** **LEMCHSTQIG** **GIRMVDILRQ** **NPTEEQAVDI** **CKAAMGLRIS** **SSFSFGGFTF** **KRTSGSSVKK** **EEEVLTGNLQ** **TLKIRVHEGY** **EEFTMVGRRA** **TAILRKATRR** **LIQLIVSGRD**

AL25/2002(II) **..........** **..........** **..........** **..........** **..........** **..........** **..........** **.........R** **..........** **...LK.....** **..........** **..........** **..........**

BKR2/2012(II) **..........** **..........** **..........** **..........** **..........** **..........** **..........** **.........R** **...M....P.** **....K.....** **.......L..** **..........** **..........**

BKR4/2012(II) **..........** **..........** **..........** **..........** **..........** **..........** **..........** **.........R** **...M......** **....K.....** **.......L..** **..........** **..........**

BKP/2012(II) **V.........** **..........** **..........** **..........** **..........** **..........** **..........** **.........R** **...M......** **....K.....** **.......L..** **..........** **..........**

AL19/2002(I) **..........** **..........** **..........** **..........** **..........** **..........** **..........** **.........R** **..........** **...LK.....** **..........** **..........** **..........**

W-04/2002(I) **.....V....** **..........** **..........** **..........** **..........** **..........** **..........** **.........S** **..........** **....K.....** **....I.....** **..........** **..........**

NWY/2012(I) **..........** **..........** **..........** **..........** **..........** **..........** **..........** **.........R** **...I......** **....K.....** **.......L..** **..........** **..........**

MAS/2013(I) **..........** **..........** **..........** **........K.** **..........** **..........** **..........** **.........R** **...I......** **....K.....** **.......L..** **..........** **..........**

**338087/2015(I)**  **..........** **..........** **..........** **..........** **..........** **..........** **..........** **.........R** **...I......** **....K.....** **.......L..** **..........** **.V........**

**341797/2015(I)**  **..........** **..........** **..........** **..........** **..........** **..........** **..........** **.........R** **...M......** **....K.....** **.......L..** **..........** **.V........**

**344378/2015(I)**  **..........** **..........** **..........** **..........** **..........** **..........** **..........** **.........R** **...I......** **....K.....** **.......L..** **..........** **.V........**

**344579/2015(I)**  **..........** **..........** **..........** **..........** **..........** **..........** **..........** **.........R** **...I......** **....K.....** **.......L..** **..........** **.V........**

**398997/2016(I)**  **..........** **..........** **..........** **..........** **..........** **..........** **..........** **.........R** **...M......** **....K.....** **.......L..** **..........** **.V........**

**401156/2016(I)**  **..........** **..........** **..........** **..........** **..........** **..........** **..........** **..........** **...M......** **....K.....** **.......L..** **..........** **.V........**

**402385/2016(I)**  **..........** **..........** **..........** **..........** **..........** **..........** **..........** **.........R** **...M......** **....K.....** **.......L..** **..........** **.V........**

**404573/2016(I)**  **..........** **..........** **..........** **..........** **..........** **..........** **..........** **.........R** **...I......** **....K.....** **.......L..** **..........** **.V........**

**N2826/2016(I)**  **..........** **..........** **..........** **..........** **..........** **..........** **..........** **.........R** **...M......** **....K.....** **.......L..** **..........** **.V........**

**H44954/2016(I)**  **..........** **..........** **..........** **..........** **..........** **..........** **..........** **.........R** **...M......** **....K.....** **....I..L..** **..........** **...M......**

**432/2019(I)**  **..........** **..........** **..........** **..........** **..........** **..........** **..........** **.........R** **...M......** **....K.....** **.......L..** **..........** **..........**

400 410 420 430 440 450 460 470 480 490 500 510 520

....|....| ....|....| ....|....| ....|....| ....|....| ....|....| ....|....| ....|....| ....|....| ....|....| ....|....| ....|....| ....|....|

KK98/1998 **EQSIAEAIIV** **AMVFSQEDCM** **IKAVRGDLNF** **VNRANQRLNP** **MHQLLRHFQK** **DAKVLFQNWG** **IEPIDNVMGM** **IGILPDMTPS** **TEMSLRGVRV** **SKMGVDEYSS** **TERVVVSIDR** **FLRVRDQRGN** **VLLSPEEVSE**

AL25/2002(II) **..........** **..........** **..........** **..........** **..........** **.......S..** **..........** **..........** **..........** **..........** **..........** **..........** **..........**

BKR2/2012(II) **..........** **..........** **..S.......** **..........** **..........** **..........** **..........** **.........N** **..........** **..........** **..........** **..........** **..........**

BKR4/2012(II) **..........** **..........** **..S.......** **..........** **..........** **..........** **..........** **.........N** **..........** **..........** **..........** **..........** **..........**

BKP/2012(II) **..........** **..........** **..S.......** **..........** **..........** **..........** **..........** **..........** **..........** **..........** **..........** **..........** **..........**

AL19/2002(I) **..........** **..........** **..........** **..........** **..........** **.......S..** **..........** **..........** **..........** **..........** **..........** **.......K..** **..........**

W-04/2002(I) .......... .......... .......... .......... .......... .......... V......... .......... .......... .......... .......... .......... ..........

NWY/2012(I) **..........** **..........** **..S.......** **..........** **..........** **..........** **..........** **..........** **..........** **..........** **..........** **..........** **..........**

MAS/2013(I) **..........** **..........** **..S.......** **..........** **..........** **N.........** **..........** **.........N** **..........** **..........** **..........** **..........** **..........**

**338087/2015(I)**  **..........** **..........** **..S.......** **..........** **..........** **..........** **..........** **V.........** **..........** **..........** **..........** **..........** **..........**

**341797/2015(I)**  **..........** **..........** **..S.......** **..........** **..........** **..........** **..........** **..........** **..........** **..........** **....I.....** **..........** **..........**

**344378/2015(I)**  **..........** **..........** **..S.......** **..........** **..........** **..........** **..........** **V.V.......** **.K........** **..........** **..........** **..........** **..........**

**344579/2015(I)**  **..........** **..........** **..S.......** **..........** **..........** **..........** **..........** **V.V.......** **..........** **..........** **..........** **..........** **..........**

**398997/2016(I)**  **..........** **..........** **..S.......** **..........** **..........** **..........** **..........** **..........** **..........** **..........** **....I.....** **..........** **..........**

**401156/2016(I)**  **..........** **..........** **..S.......** **..........** **..........** **..........** **..........** **..........** **..........** **..........** **....I.....** **..........** **..........**

**402385/2016(I)**  **..........** **..........** **..S.......** **..........** **..........** **..........** **..........** **..........** **..........** **..........** **....I.....** **..........** **..........**

**404573/2016(I)**  **..........** **..........** **..S.......** **..........** **..........** **..........** **V.....I...** **..........** **..V.......** **..T.......** **....I.....** **..........** **..........**

**N2826/2016(I)**  **..........** **..........** **..S.......** **..........** **..........** **..........** **..........** **..........** **..........** **..........** **....I.....** **..........** **..........**

**H44954/2016(I)**  **..........** **..........** **..S.......** **..........** **..........** **..........** **..........** **..........** **..........** **..........** **....I.....** **..........** **..........**

**432/2019(I)**  **D.........** **..........** **..S.......** **..........** **..........** **..........** **..........** **..........** **..........** **..........** **....I.....** **..........** **..........**

530 540 550 560 570 580 590 600 610 620 630 640 650

....|....| ....|....| ....|....| ....|....| ....|....| ....|....| ....|....| ....|....| ....|....| ....|....| ....|....| ....|....| ....|....|

KK98/1998 **TQGTEKLTIT** **YSSFMMWEIN** **GPESVLVNTY** **QWIIRNWETV** **KIQWSQDPTM** **LYNKMEFEPF** **QSLVPKAARG** **QYSGFVRTLF** **QQMRDVLGTF** **DTVQIIKLLP** **FAAAPPEQSR** **MQFSSLTVNV** **RGSGMRILVR**

AL25/2002(II) **..........** **...S......** **..........** **..........** **..........** **..........** **..........** **..........** **..........** **....L.....** **..........** **..........** **..........**

BKR2/2012(II) **..........** **...S......** **..........** **..........** **..........** **..........** **..........** **.......A..** **..........** **..........** **..........** **..........** **..........**

BKR4/2012(II) **..........** **...S......** **..........** **..........** **..........** **..........** **..........** **.......A..** **..........** **..........** **..........** **..........** **..........**

BKP/2012(II) **..........** **...S......** **..........** **..........** **..........** **..........** **..........** **.......A..** **..........** **..........** **..........** **..........** **..........**

AL19/2002(I) **..........** **...S......** **..........** **..........** **..........** **..........** **..........** **..........** **..........** **....L.....** **..........** **..........** **..........**

W-04/2002(I) **.....R....** **...S......** **..........** **..........** **..........** **..........** **..........** **..........** **..........** **....L.....** **..........** **.........I** **..........**

NWY/2012(I) **..........** **...S......** **..........** **..........** **..........** **..........** **..........** **.......A..** **..........** **..........** **......G...** **..........** **..........**

MAS/2013(I) **..........** **...S......** **..........** **..........** **..........** **..........** **..........** **H......A..** **..........** **..........** **......G...** **..........** **..........**

**338087/2015(I)**  **..........** **...S......** **..........** **..........** **..........** **..........** **..........** **.......A..** **..........** **..........** **..........** **..........** **..........**

**341797/2015(I)**  **..........** **...S......** **......I...** **..........** **........AV** **..........** **..........** **.......A..** **..........** **..........** **..........** **..........** **....V.....**

**344378/2015(I)**  **..........** **...S......** **..........** **..........** **..........** **..........** **..........** **.......A..** **..........** **..........** **..........** **..........** **..........**

**344579/2015(I)**  **..........** **...S......** **..........** **..........** **..........** **..........** **..........** **.......A..** **..........** **..........** **..........** **..........** **..........**

**398997/2016(I)**  **..........** **...S......** **......I...** **..........** **........AV** **..........** **..........** **.......A..** **..........** **..........** **..........** **..........** **....V.....**

**401156/2016(I)**  **..........** **...S......** **......I...** **..........** **........AV** **..........** **..........** **.......A..** **..........** **..........** **..........** **..........** **....V.....**

**402385/2016(I)**  **..........** **...S......** **......I...** **..........** **........AV** **..........** **..........** **.......A..** **..........** **..........** **..........** **..........** **....V.....**

**404573/2016(I)**  **..........** **...S......** **......I...** **..........** **........AV** **..........** **..........** **.......A..** **..........** **..........** **..........** **..........** **....V.....**

**N2826/2016(I)**  **..........** **...S......** **......I...** **..........** **........AV** **..........** **..........** **.......A..** **..........** **..........** **.T........** **..........** **....V.....**

**H44954/2016(I)**  **..........** **.L.S......** **..........** **..........** **..........** **..........** **.......T..** **.......A..** **..........** **..........** **..........** **......I...** **..........**

**432/2019(I)**  **..........** **...S......** **......I...** **..........** **........AV** **..........** **..........** **..........** **..........** **..........** **..........** **..........** **....V.....**

660 670 680 690 700 710 720 730 740 750 760

....|....| ....|....| ....|....| ....|....| ....|....| ....|....| ....|....| ....|....| ....|....| ....|....| ....|....|

KK98/1998 **GNSPVFNYNK** **ATKRLTVLGK** **DAGALTEDPD** **EGTAGVESAV** **LRGFLILGKE** **DRRYGPALSI** **NELSNLAKGE** **KANVLIGQGD** **VVLVMKRKRD** **SSILTDSQTA** **TKRIRMAIN***

AL25/2002(II) **.........R** **..........** **..........** **..A.......** **..........** **..........** **..........** **..........** **..........** **..........** **.........***

BKR2/2012(II) **.........R** **..........** **..........** **...V......** **..........** **.K........** **..........** **..........** **..........** **.......H..** **.........***

BKR4/2012(II) **.........R** **..........** **..........** **...V......** **..........** **.K........** **..........** **..........** **..........** **..........** **.........***

BKP/2012(II) **.........R** **..........** **..........** **...V......** **..........** **.K........** **..........** **..........** **..........** **..........** **.........***

AL19/2002(I) **.........R** **..........** **..........** **..A.......** **..........** **N.........** **..........** **..........** **..........** **.......H..** **.........***

**W**-04/2002(I) **.........R** **..........** **..........** **..A.......** **..........** **..........** **..........** **..........** **..........** **.......H..** **.........***

NWY/2012(I) **.........R** **..........** **..........** **..........** **..........** **.K........** **......V...** **..........** **..........** **..........** **.........***

MAS/2013(I) **.........R** **..........** **..........** **..........** **..........** **.K........** **......V...** **..........** **..........** **..........** **.........***

**338087/2015(I)**  **.........R** **..........** **..........** **..........** **..........** **.K........** **......V...** **..........** **..........** **..........** **.........***

**341797/2015(I)**  **.........R** **......I...** **..........** **..........** **..........** **..........** **..........** **..........** **..........** **..........** **.........***

**344378/2015(I)**  **.........R** **..........** **..........** **..........** **..........** **.K........** **......V...** **..........** **..........** **..........** **.........***

**344579/2015(I)**  **.........R** **..........** **..........** **..........** **..........** **.K........** **......V...** **..........** **..........** **..........** **.........***

**398997/2016(I)**  **.........R** **......I...** **..........** **..........** **..........** **..........** **S.........** **..........** **..........** **..........** **.........***

**401156/2016(I)**  **.........R** **......I...** **..........** **..........** **..........** **..........** **..........** **..........** **..........** **..........** **.........***

**402385/2016(I)**  **.........R** **......I...** **..........** **..........** **..........** **..........** **..........** **..........** **..........** **..........** **.........***

**404573/2016(I)**  **.........R** **......I...** **..........** **..........** **..........** **..........** **..........** **..........** **..........** **..........** **.........***

**N2826/2016(I)**  **.........R** **......I...** **..........** **..........** **..........** **..........** **..........** **..........** **..........** **..........** **.........***

**H44954/2016(I)**  **.........R** **......I...** **..........** **..........** **..........** **..........** **..........** **..........** **..........** **..........** **.........***

**432/2019(I)**  **.........R** **......I...** **....M.....** **..........** **..........** **..........** **..........** **..........** **..........** **..........** **.........***
